# From Nature to Nanomedicine: Enhancing the Antitumor Efficacy of Rhein, Curcumin, and Resveratrol

**DOI:** 10.3390/medicina61060981

**Published:** 2025-05-26

**Authors:** Ana-Maria Trofin, Dragoș Viorel Scripcariu, Silviu-Iulian Filipiuc, Anca-Narcisa Neagu, Leontina-Elena Filipiuc, Bogdan-Ionel Tamba, Madalina Maria Palaghia, Cristina Mariana Uritu

**Affiliations:** 1 Department of Surgery, Faculty of Medicine, “Grigore T. Popa” University of Medicine and Pharmacy, 16 Universitatii Street, 700115 Iasi, Romania; trofin_ana_maria@yahoo.com (A.-M.T.);; 2 Surgery Clinic, “Sf Spiridon” Emergency County Hospital, 700111 Iasi, Romania; 3 1st Surgical Oncology Unit, Regional Institute of Oncology, 2–4 General Henri Mathias Berthelot Street, 700483 Iasi, Romania; 4 Advanced Center for Research and Development in Experimental Medicine “Prof. Ostin C. Mungiu”, “Grigore T. Popa” University of Medicine and Pharmacy, 700115 Iasi, Romaniacristina-mariana.uritu@umfiasi.ro (C.M.U.); 5 Department of Physiology, “Grigore T. Popa” University of Medicine and Pharmacy, 16 Universitatii Street, 700115 Iasi, Romania; 6 Laboratory of Animal Histology, Faculty of Biology, “Alexandru Ioan Cuza” University of Iasi, Carol I bvd, No. 20A, 700505 Iasi, Romania; 7 Department of Pharmacology, Clinical Pharmacology and Algesiology, “Grigore T. Popa” University of Medicine and Pharmacy, 16 Universitatii Street, 700115 Iasi, Romania

**Keywords:** curcumin, resveratrol, rhein, bioactive natural substances in cancer therapy, nanodelivery systems

## Abstract

Natural compounds have garnered increasing interest as potential antitumor agents due to their multifaceted biological activities and relatively low toxicity profiles. This review focuses on three well-studied natural molecules: rhein, curcumin, and resveratrol, analyzing and comparing their antitumor potential across a variety of cancer models. For each compound, we present an integrated perspective encompassing natural sources, physicochemical properties, pharmacological and pharmacokinetic characteristics, and the latest in vitro and in vivo evidence of anticancer activity. Special attention is given to the molecular mechanisms underlying their antitumor effects, including the modulation of cell cycle regulators, induction of apoptosis, inhibition of metastasis and angiogenesis, and regulation of key signaling pathways such as NF-κB, PI3K/Akt/mTOR, STAT3, and MAPKs. Although numerous studies highlighted their therapeutic promise, significant barriers remain—particularly related to poor solubility and limited bioavailability—which have hindered clinical translation, especially in the case of rhein. Advances in nanotechnology-based drug delivery systems offer promising solutions to these limitations, enabling improved targeting and enhanced efficacy. This review underscores the need for continued preclinical and clinical investigations to fully elucidate the therapeutic value of these compounds and support their integration into modern oncological treatment strategies.

## 1. Introduction

Despite the impressive advances in oncology recorded in recent years, the exploration of potentially revolutionary therapeutic approaches needs to continue due to the limitations of conventional cancer treatments, such as the development of resistance to chemotherapy, high toxicity including for healthy tissues, and the risk of relapse [1,2,3]. Natural compounds have emerged as highly attractive alternatives for cancer therapy due to their extensive biological activity, favorable safety profiles, and ability to target multiple molecular pathways. Nonetheless, the implementation of natural products into clinical oncology requires an exhaustive evaluation of their real effectiveness as therapeutics, either as a stand-alone treatment or as an adjuvant to current therapy, along with conventional antitumor drugs.

Historically, several of the most effective anticancer agents in clinical practice today have originated as natural plant extracts. Early observations of biological activity in traditional medicine led to the scientific isolation and characterization of key compounds such as paclitaxel, derived from *Taxus brevifolia*; vincristine and vinblastine, from *Catharanthus roseus*; and etoposide, a semi-synthetic derivative of podophyllotoxin extracted from *Podophyllum peltatum* [4,5,6]. Initially regarded merely as promising cytotoxic substances, these natural molecules underwent rigorous preclinical and clinical validation, eventually becoming essential components of modern chemotherapy regimens and achieving gold standard status in the treatment of cancers such as breast cancer, ovarian cancer, leukemia, and testicular cancer. This historical success of natural products in oncology underscores the immense therapeutic potential inherent in bioactive compounds of natural origin. It also provides a strong rationale for ongoing research into other phytochemicals with demonstrated antitumor properties.

Among the numerous bioactive molecules investigated, curcumin, resveratrol, and rhein have gained particular interest due to their demonstrated antitumor properties in various preclinical models and emerging evidence from clinical studies [7,8,9]. Although structurally distinct, these compounds share common mechanisms, such as modulation of oxidative stress, inhibition of inflammatory pathways, induction of apoptosis, and interference with cell proliferation and metastasis [10,11,12,13,14,15,16,17,18,19,20,21,22,23,24,25]. Understanding whether these agents can evolve beyond adjunctive roles to become primary therapeutic options requires a critical evaluation of their mechanisms of action, pharmacokinetic limitations, and recent advances in formulation strategies designed to enhance their clinical efficacy. However, despite their promising biological activities, the clinical application of curcumin, resveratrol, and rhein faces significant limitations, primarily due to their poor aqueous solubility, low bioavailability, rapid metabolism, and systemic elimination [26]. These pharmacokinetic drawbacks severely restrict the effective delivery of therapeutic concentrations at the tumor site and diminish their clinical efficacy. Consequently, considerable research efforts have been directed toward the development of advanced drug delivery systems, such as nanoparticles, liposomes, polymeric carriers, and other nanotechnological approaches, aiming to enhance the stability, solubility, and targeted delivery of these natural compounds [9,26,27].

The first objective of this article is to perform a comparative analysis of the available preclinical and clinical data on curcumin, resveratrol, and rhein, with a focus on their shared and distinct anticancer mechanisms of action. Additionally, we aim to critically assess whether these compounds demonstrate sufficient efficacy to be considered as independent therapeutic agents or whether their role is better suited as adjuncts to conventional oncologic treatments. Through this approach, we seek to provide a clearer understanding of the current evidence and future perspectives concerning the integration of natural compounds into cancer management strategies. We will also explore the most recent strategies employed to overcome the key pharmacokinetic challenges, emphasizing the role of innovative formulation techniques in maximizing the therapeutic potential of curcumin, resveratrol, and rhein in cancer management.

This article aims to contribute to the ongoing evaluation of curcumin, resveratrol, and rhein by providing an integrated perspective on their antitumor potential, drawing on both historical insights and the most recent advances in biomedical research, with a focus on their mechanisms of action, therapeutic positioning, and formulation-related challenges.

## 2. Antitumor Properties of Natural Compounds

### 2.1. Rhein

#### 2.1.1. Natural Source, Chemical Properties, and Therapeutic Potential of Rhein

**Figure 1:**
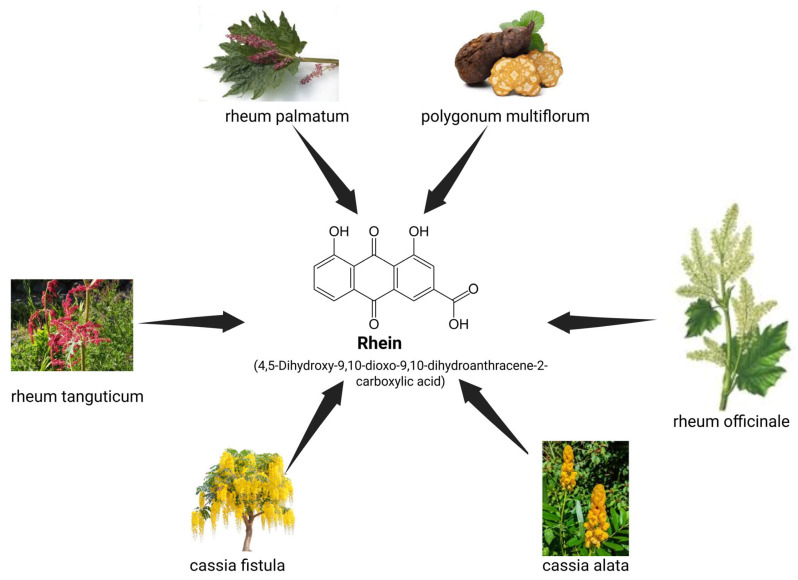
Natural sources of rhein.

Rhein (1,8-dihydroxy-3-carboxy anthraquinone, C_15_H_8_O_6_) is an anthranoid compound principally obtained from medicinal plants belonging to the genus *Rheum* (rhubarb), particularly *Rheum palmatum*, *Rheum officinale*, and *Rheum tanguticum* [28]. This bioactive compound is also found in significant quantities in *Cassia species*, including *Cassia fistula* (golden shower tree) and *Cassia alata* (Figure 1) [29]. Additionally, *Aloe* species contain rhein as a secondary metabolite, as does the plant *Polygonum multiflorum* [30]. Some plant-derived traditional medicines, particularly in Chinese, Indian, and Korean pharmacopeia, contain rhein as an active anthraquinone derivative. The compound can also be synthesized chemically through various routes, including oxidation of aloe-emodin or condensation reactions between phthalic anhydride derivatives and resorcinol followed by oxidation.

Rhein is a crystalline compound appearing as a yellow-orange powder with a molecular weight of 284.22 g/mol. It exhibits poor water solubility (lower than 0.1 mg/100 mL at 17 °C) but demonstrates higher solubility in polar organic solvents such as ethanol, methanol, and DMSO [31]. Rhein possesses two phenolic hydroxyl groups at positions C-1 and C-8 and a carboxylic acid group at position C-3, contributing to its acidic properties (pKa ≈ 4.5) [32]. The compound displays characteristic UV absorption maxima at approximately 228, 257, and 430 nm in methanol, and also fluorescence emission with maxima typically observed between 540–580 nm when excited at wavelengths between 430 and 450 nm, yielding a yellow-green fluorescence [33]. The fluorescence properties rely on the conjugated anthraquinone structure, with two hydroxyl groups at positions C-1 and C-8. It should be mentioned that under alkaline conditions (pH > 8), rhein exhibits a distinct red-violet color due to the ionization of its phenolic groups, showing an enhanced fluorescence emission and also bathochromic shifts in both excitation and emission spectra [34]. These features are very important as sensitive spectrometric methods for rhein detection and quantification in biological samples or pharmaceutical formulations [26].

The anthraquinone core structure of rhein proves significant stability, though the compound is sensitive to oxidation and UV degradation in solution [35]. NMR spectra have confirmed its planar configuration with intramolecular hydrogen bonding between the hydroxyl and adjacent carbonyl groups [36].

Since rhein originates from traditional Chinese medicinal herbs, it has been widely used in traditional medicine for centuries due to its broad therapeutic profile [37]. Habitually, rhein has been employed as a natural laxative, liver tonic, and remedy for infections and inflammatory conditions [37]. Recent studies support its strong anti-inflammatory, antioxidant, antimicrobial, antiviral, anti-fibrotic, cardio-cerebral protection, hepato- and nephroprotective properties [38,39,40,41,42,43,44,45,46]. It has demonstrated efficacy in models of diabetic nephropathy, where it reduces renal inflammation and fibrosis, and in liver injury, where it prevents oxidative damage and enhances detoxification pathways. Rhein also exhibits potential benefits in cardiovascular disorders by improving endothelial function and reducing lipid accumulation. Additionally, its antimicrobial effects include activity against various Gram-positive and Gram-negative bacteria, supporting its historical use for treating infections and gastrointestinal disturbances. These findings validate rhein’s traditional uses and highlight its relevance for chronic inflammatory and metabolic conditions.

Rhein demonstrates complex pharmacokinetic behavior that varies significantly across different administration routes. When administered orally, rhein exhibits relatively poor bioavailability (approximately 10–30%) due to several limiting factors, including its poor water solubility, extensive first-pass metabolism in the liver, and efflux by P-glycoprotein transporters in the intestinal epithelium [37]. Absorption primarily occurs in the small intestine, with peak plasma concentrations typically observed 1.5–2 h post-administration [47]. The compound undergoes significant enterohepatic circulation, with glucuronidation as the primary metabolic pathway [48].

Parenteral administration (intravenous, subcutaneous, or intramuscular) of rhein formulations circumvents first-pass metabolism, resulting in substantially higher bioavailability (>85%) [49]. However, parenteral delivery presents challenges due to rhein’s poor water solubility and potential for injection site reactions. Various drug delivery systems including liposomes, nanoparticles, and cyclodextrin complexes have been investigated to improve parenteral delivery.

Although the systemic bioavailability of rhein following topical application remains limited, its pronounced hydrophobicity facilitates a strong affinity for lipid-rich skin layers, leading to significant skin retention. In vitro permeation studies confirmed rhein’s ability to accumulate in the skin and achieve the highest receptor-phase concentration among the tested anthraquinones, supporting its potential for local therapeutic action and controlled transdermal delivery [50]. Transdermal delivery can be enhanced through the use of penetration enhancers or specialized delivery systems like microemulsions or ethosomal carriers.

#### 2.1.2. Antitumor Mechanisms of Rhein

Evidence suggests that rhein and its derivatives have anticancer activities in various malignancies, such as breast cancer (BC) overexpressing HER2/neu (MCF7/HER2), triple-negative BC (MDA-MB-231 and 4T1 TNBC cell lines), oral cancer (OC) (YD-10B and Ca9-22 cell lines), gastric cancer (HGC-27), liver cancer (HepG2 and Huh7), colorectal cancer (CRC), ovarian cancer (A2780, SKOV3, SKOV3-PM), and non-small-cell lung cancer [10,11,18,19,51,52,53,54,55,56].

Rhein and its derivatives exert antiproliferative effects [10,18,52,54]. Rhein induces apoptosis in dose- and time-dependent manners, by increasing caspase-9-mediated or caspase-3-mediated apoptosis [10,18,52,53]. In addition, the rhein-4a derivative induces paraptosis-like cell death mechanisms, as an alternative, non-apoptotic form of programmed cell death, characterized by endoplasmic reticulum (ER) swelling and fusion, mitochondria damage, cytoplasmic vacuolization, and the non-involvement of caspase activation [55]. Rhein and its derivatives inhibit migration and invasion through the epithelial-to-mesenchymal transition (EMT) [18,19,54].

Multiple investigations exploring the anticancer effects of rhein aimed to establish its mechanism of action. Rhein induces suppression of the AKT/mTOR signaling pathway in vitro and in vivo, by mTOR degradation through the ubiquitin–proteasome pathway [18,19]. Moreover, rhein modulates the activation of the NF-kB and p53 signaling pathways and influences the NOTCH/JAK/STAT signaling pathway [10,52]. Rhein and its derivatives (4F) also cause cytoskeletal changes like those caused by paclitaxel [51]. Additionally, rhein downregulates RAC family small GTPase 1 (RAC1) expression involved in malignant behavior and is a promising therapeutic target in BC [51]. In ovarian cancer cell lines, the 4a derivative of rhein induced activation of the PERK-eIF2α-ATF4 pathways, correlated with the activation of p38, ERK, and JNK and downregulation of ALIX, a protein that inhibits paraptosis [55].

Compared to rhein, the 4F derivative, obtained by the introduction of a benzyloxy group at the 1,8-phenolic hydroxyl group, which induces endoplasmic reticulum stress, as well as a hydroxyethyl piperazine group to the 3-carboxyl group, more strongly inhibited BC cell proliferation, migration, and invasion [51]. The 4a rhein derivative (1,8-Bis(benzyloxy)-9,10-anthraquinone-N-(2-hydroxyethyl)-3-carboxamide) was obtained by introducing a bisbenzyloxy group at the 1,8-OH position of rhein, modifying and structurally optimizing it at the C-3 position, resulting in obvious antitumor activity and many cytoplasmic vacuoles in ovarian cancer cells [55].

In the following, we outline specific examples of rhein’s mechanisms of action, organized by cancer type, in order to illustrate the molecular pathways involved and the corresponding cellular or in vivo effects reported in the literature.

Rhein activates NF-κB and the p53/p21 pathway via ASK1 in HER2-positive MCF7/HER2 BC cells, resulting in antiproliferative and pro-apoptotic effects [10]. In triple-negative BC, treatment with a rhein derivative (4F) leads to cytoskeletal changes through RAC1 inhibition and downregulation, suppressing the proliferation, migration, and invasion of MDA-MB-231 cells [18]. Moreover, HA liposomal delivery of rhein in 4T1 breast-cancer-bearing mice enhances tumor targeting and cytotoxicity, significantly inhibiting distant metastases [11].

In **oral cancer** cell lines YD-10B and Ca9-22, rhein suppresses the AKT/mTOR signaling pathway and inhibits the epithelial–mesenchymal transition, as evidenced by increased E-cadherin and reduced N-cadherin expression. These molecular alterations promote apoptosis, inhibit cell proliferation, trigger ROS generation, suppress autophagy, and reduce cell motility and invasiveness [18].

In HGC-27 **gastric cancer** cells, rhein interferes with the NOTCH/JAK/STAT axis by downregulating Bcl-2, JAK1, JAK2, and STAT3. This leads to marked antiproliferative and pro-apoptotic activity, confirming its anticancer potential in this context [52].

Studies on HepG2 and Huh7 hepatic cancer cells show that rhein activates the ROS/JNK/c-Jun/caspase-3 signaling cascade, in a dose- and time-dependent manner, causing the loss of mitochondrial membrane potential [53]. The resulting oxidative stress and apoptotic signaling reduce cell viability and proliferation [53].

In **colorectal cancer models**, including HCT15, HCT116, HT29, and SW620 cell lines and HCT116 xenografts, rhein inhibits mTOR signaling and the epithelial–mesenchymal transition. It induces degradation of mTOR via the ubiquitin–proteasome pathway and downregulates cyclin A1/E1, CDK1, and N-cadherin while upregulating E-cadherin. The expression of p-mTOR, p-p70S6K, HSF1, and cyclin D1 is also decreased in tumor tissues, contributing to reduced proliferation, increased apoptosis, and inhibition of migration and invasion [19].

In **ovarian cancer**, rhein inhibits matrix metalloproteinases (MMPs) in A2780 and OV2008 cells, thereby reducing cell proliferation and migration [54]. Additionally, treatment of SKOV3, SKOV3-PM, and A2780 cells with the 4a rhein derivative activates the PERK-eIF2α-ATF4 axis and the MAPK cascade (p38, ERK, JNK) while suppressing ALIX expression. This induces cell cycle arrest and a paraptosis-like form of cell death marked by endoplasmic reticulum swelling, organelle fusion, mitochondrial damage, and activation of the unfolded protein response [55]. It was observed that rhein induces a dose-dependent inhibition of cell migration in both parental and resistant AML cells. Inhibition of the AKT/mTOR pathway by rhein can effectively suppress the migratory potential of AML cells. Rhein has shown dose-dependent effects on proliferation and migration in two typical ovarian cancer cell lines, A2780 and OV2008. MTT assays showed that the ovarian cancer cell survival rate decreased significantly after rhein treatment. In addition, wound healing and transwell assays indicated a clear inhibition of cell migration and matrix metalloproteinase expression [54].

In **non-small-cell lung cancer models** (PC-9, H460, A549), rhein inhibits the STAT3/ Snail/MMP2/MMP9 signaling pathway, upregulates the pro-apoptotic protein Bax, and downregulates the anti-apoptotic factor Bcl-2. These molecular changes induce cell cycle arrest and apoptosis, reduce viability, and suppress tumor growth in H460 xenograft models [20,56].

Studies have shown that rhein can promote apoptosis in the human liver cell line KL-7702 and the human tongue squamous cell carcinoma line SCC-4 by affecting endoplasmic reticulum stress and caspase- or mitochondria-dependent pathways [57].

#### 2.1.3. Preclinical Applications and Clinical Trials

In a study on tumor-bearing mice receiving low (75 mg/kg) or high (150 mg/kg) intraperitoneal doses of rhein, it was observed that rhein could inhibit tumor growth without other tissue toxicities due to restrictive conditions [58]. Rhein’s bioavailability is a key factor in its in vivo efficacy. A study showed that rhein exerts antitumor effects in RCC cells by inactivating the MAPK/NF-κB signaling pathways. Two previous studies demonstrated that rhein inhibits tumorigenesis by suppressing MAPK phosphorylation [59]. Most interestingly, it was found that rhein significantly sensitizes cells to erlotinib, thus suppressing tumor growth in PANC-1 and BxPC-3 xenograft models. The in vivo antitumor effect was associated with increased apoptosis and the combined inhibition of the STAT3 and EGFR pathways. Rhein sensitizes human pancreatic cancer cells to EGFR inhibitors by inhibiting STAT3, providing an innovative framework for pancreatic cancer treatment, especially in combination with EGFR inhibitors [60].

Studies suggest that rhein has anticancer effects on gastric cancer. Rhein inhibited the viability of MGC-803 cells in a dose-dependent manner (IC50 = 94.26 μM). Everolimus had a similar effect (IC50 = 45.41 nM). The rhein–everolimus combination significantly suppressed viability, invasion, and cell proliferation compared to individual administration. The combination also facilitated apoptosis and increased expression of proteins associated with apoptosis and the cell cycle (p53, CDK4, cyclin D1). At the same time, it suppressed the expression of phospho-PI3K, phospho-AKT, and phospho-mTOR. Ultimately, the combination reduced tumor weight and volume and decreased the expression of the Ki-67 proliferation marker, demonstrating synergistic prevention against gastric cancer via the PI3K/Akt/mTOR pathway [61].

Another study found that a nontoxic concentration of rhein reversed TRAIL (tumor necrosis factor-related apoptosis-inducing ligand) resistance and increased TRAIL-mediated apoptosis in bladder cancer cells by increasing DR5 expression. This combination effectively enhanced TRAIL-induced apoptosis in bladder cancer cells without being cytotoxic to normal bladder epithelium cells, suggesting its potential clinical value for bladder cancer treatment [62].

It has been demonstrated that rhein can restore chemosensitivity in the SMMC-7721 liver cancer cell line to doxorubicin and in pancreatic cancer cells to EGFR inhibitors by inhibiting mitochondrial energy metabolism and the STAT3 pathway, respectively [21,60].

Synergistic antiproliferative and pro-apoptotic effects were exerted by combining rhein (10 μM) and DOX (2 μM) in SMMC-7721 and HepG2 cells. Rhein influenced the accumulation of DOX in both cell types, an effect associated with a significant decrease in mitochondrial energy metabolism and ATP levels. Rhein also reduced the mitochondrial membrane potential (ΔΨm) in both cell lines. Opening of the mitochondrial permeability transition pore (mPTP) with atractyloside (ATR) accelerated the loss of mitochondrial membrane potential (ΔΨm) and further reduced the oxygen consumption rate (OCR) induced by rhein. In contrast, the mPTP blocker cyclosporin A (Cs A) inhibited the loss of ΔΨm and the rhein-induced OCR. The data showed that the decrease in mitochondrial energy metabolism is responsible for the synergistic antitumor effects of the rhein–DOX combination in hepatocellular carcinoma cells. The reduction in ΔΨm and the opening of mPTP, which inhibit the exchange of ATP/adenosine diphosphate between the mitochondrial matrix and the cytoplasm, represent a key mechanism [63].

### 2.2. Curcumin

#### 2.2.1. Natural Source, Chemical Properties, and Therapeutic Potential of Curcumin

**Figure 2:**
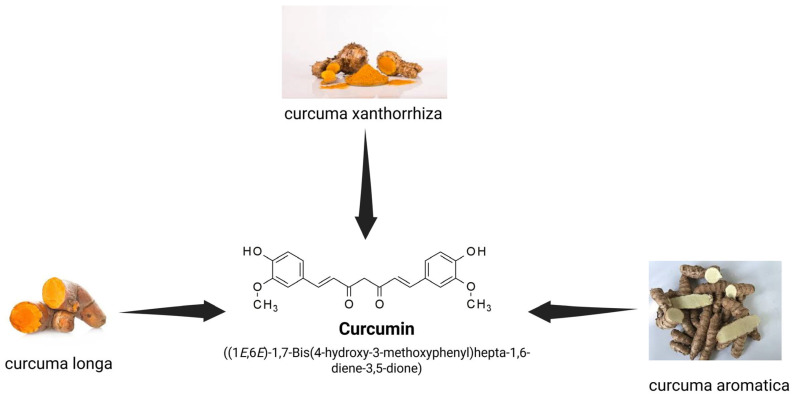
Natural sources of curcumin.

Curcumin is a natural hydrophobic compound (1,7-bis(4-hydroxy-3-methoxyphenyl)-1,6-heptadiene-3,5-dione), biosynthesized in about 130 species of *Curcuma*, including, among others, *Curcuma longa* (turmeric), *Curcuma aromatica* (wild turmeric), and *Curcuma xanthorrhiza* (Javanese turmeric) (Figure 2) [7]. Curcumin is produced in the rhizomes of these plants through a complex enzymatic process that starts with phenylalanine ammonia-lyase’s conversion of phenylalanine to cinnamic acid. Curcuminoids, such as curcumin, demethoxycurcumin, and bis-demethoxycurcumin, are synthesized through intermediate stages, with p-coumaric acid serving as an important precursor [7,64]. *Curcuma longa* (fam. *Zingiberaceae*), the most important source of curcumin, has been used in traditional Indian, Chinese, and Arabic medicine for over four millennia, and it is recognized for its multiple therapeutic properties. Turmeric has been used in these traditional medical systems to treat a wide range of conditions, such as respiratory disorders (like asthma, bronchial hyper-reactivity, and common colds), ENT disorders (sinusitis), liver and metabolic diseases (like cirrhosis, fatty liver disease, hepatitis, metabolic syndrome, type 2 diabetes, and dyslipidemia), and rheumatic and dermatological conditions [1,3,4,5]. It was also used to treat diabetic wounds, to reduce inflammation in chronic inflammatory disorders, and as an adjuvant in the treatment of anorexia [7,27,65,66].

With its particular phenolic groups and interconnected bonds, the unusual chemical structure of curcumin enables it to have several effects: it can neutralize free radicals, modify cell signaling pathways, and bind to metal particles in the body. Due to its lipophilic nature, curcumin has a low solubility in water, which significantly limits the oral bioavailability. However, experimental studies have revealed a remarkable therapeutic potential. Curcumin acts as an antioxidant, neutralizing free radicals and protecting cells from oxidative stress. Additionally, it has a pronounced anti-inflammatory effect, by inhibiting proinflammatory signaling pathways, such as NF-κB and COX-2, thus contributing to the reduction in systemic and local inflammation [7,27]. Its hepatoprotective, cardioprotective, and immunomodulatory properties have been confirmed in numerous preclinical models [67,68,69,70,71]. Moreover, curcumin has demonstrated neuroprotective effects by reducing neuronal inflammation, preventing cellular degeneration, and stimulating neurogenesis, suggesting valuable potential in the treatment of neurodegenerative diseases [72]. Finally, curcumin also exhibits antitumor activity. It has been extensively studied for its ability to inhibit the proliferation of cancer cells, trigger apoptosis in abnormal cells, and enhance the effectiveness of conventional cancer therapies in various malignancies, including colorectal and pancreatic cancers [7,27,73].

All these effects support the idea that, despite limitations related to stability and bioavailability, curcumin remains a promising candidate for the development of innovative therapies, especially through the use of modern delivery systems that maximize its therapeutic potential.

#### 2.2.2. Antitumor Mechanisms of Curcumin

Curcumin exerts its anticancer activities through a variety of molecular and cellular pathways that work together to suppress tumor initiation, development, and metastasis. These mechanisms include the inhibition of proinflammatory transcription factors, the modulation of oncogenic signaling pathways, the inhibition of angiogenesis and metastasis, the modulation of gene expression via epigenetic modifications, the alteration of cellular and mitochondrial metabolism, and interfering with the tumor immune environment [74]. The main pathways through which curcumin exerts its antineoplastic effect are discussed in detail below.

Curcumin exerts a major antitumor effect by suppressing cell proliferation and activating cell death mechanisms. In this regard, curcumin blocks the progression of the cell cycle at the transition between the second gap phase (G2 phase) and mitosis by downregulating cyclins and cyclin-dependent kinases (CDKs) and increasing the expression of their inhibitors [75,76,77]. Apoptosis is triggered by intrinsic and extrinsic pathways, involving the activation of pro-apoptotic proteins (Bim, Bax, Bak, PUMA, or Noxa) and cell death receptors (TRAIL-R1/R2), along with the downregulation of anti-apoptotic proteins such as Bcl-2, Bcl-XL, survivin (BIRC5), and XIAP [78,79,80]. These changes lead to the release of cytochrome c and the activation of caspases, initiating programmed cell death [22].

Seong-Su Han et al. observed that at low concentrations, curcumin more effectively inhibits the proliferation of immature B cells (BKS-2) than normal ones by inducing apoptosis through downregulation of survival genes (EGR-1, c-myc, Bcl-XL) and the tumor suppressor gene p53 [23]. Lichuan Wu et al. highlighted that curcumin inhibits proliferation and colony formation in the NCI-H460 **lung cancer** cell line by inhibiting the JAK2/STAT3 pathway [81]. Furong Liu et al. demonstrated that in A549 NSCLC cells, curcumin inhibits the PI3K/Akt/mTOR pathway, inducing apoptosis and autophagy [24]. Jia Rao et al. showed a similar effect in daunorubicin-resistant AML CD34+ cells, revealing that curcumin reduced Bcl-2 expression and mitochondrial membrane potential loss and activated caspase-3 and PARP [82]. Curcumin has been reported to suppress the proliferation of several **breast cancer cell lines** (MDA-MB-231, MDA-MB-468, T47D, MCF-7) with a median inhibitory concentration (IC50) in the micromolar range. Its mechanism of action involves cell cycle arrest in the G2/M phase by reducing the expression of CDC2 and CDC25 proteins and increasing p21 expression [83].

Curcumin disrupts important communication networks inside cells that contribute to cancer development, especially pathways known as PI3K/Akt/mTOR and MAPK/ERK [74,84]. By preventing certain proteins from being activated through a process called phosphorylation, curcumin slows down how quickly cancer cells multiply and helps trigger apoptosis [85]. The suppression of the MAPK/ERK cascade also contributes to blocking the differentiation and invasion of cancer cells [84].

Curcumin suppresses transcription factors involved in tumor survival and proliferation, especially nuclear factor kappa B (NF-κB), STAT3, and AP-1 [86,87]. Specifically, curcumin decreases the expression of these pro-oncogenic transcriptional activators not only through direct action but also by inhibiting kinases associated with their activation, such as IκKβ [88]. This dual action contributes to the suppression of transcription of genes involved in inflammation, uncontrolled proliferation, and tumor resistance, reinforcing its antineoplastic effect.

Gizem Calibasi-Kocal et al. emphasized curcumin’s dose-dependent chemoprotective role in **colon cancer cells** HCT-116 and LoVo by inhibiting NF-κB and activating caspases 3 and 9 [89].

Curcumin exerts anti-angiogenic effects by reducing the expression of VEGF and its receptor VEGFR2, blocking the formation of new blood vessels [90,91]. In addition, it inhibits metastasis by downregulating the metalloproteinases MMP-2 and MMP-9, essential in the degradation of the extracellular matrix, and by disrupting the CXCL12/CXCR4 chemokine axis involved in the epithelial–mesenchymal transition and tumor cell migration [12,25].

Curcumin acts on several modulators of the immune system, which indirectly contributes to its antitumor effects. It regulates the expression of proinflammatory cytokines, such as TNF-α, interleukins (IL-1, IL-2, IL-6, IL-8, IL-12), and the chemokine cyclooxygenase-2 (COX-2), molecules involved in tumor initiation and progression [13]. By inhibiting these factors, curcumin contributes to a reduction in chronic inflammation, a key process in carcinogenesis.

Reactive oxygen species (ROS) can act as secondary messengers in cell signaling pathways involved in cell proliferation, survival, and differentiation. Curcumin has the ability to directly bind to free radicals, acting as an effective “scavenger”, which contributes to limiting oxidative stress and, implicitly, reducing tumor growth and metastatic potential [66,92].

Curcumin enhances the immune response by modulating the activity of various immune cells, including B and T lymphocytes, macrophages, and dendritic cells. One notable effect is its ability to inhibit the maturation of dendritic cells by downregulating the expression of the co-stimulatory molecules DC80 and DC86 [93,94]. In addition, curcumin suppresses the production of proinflammatory cytokines, thereby contributing to the creation of a tumor microenvironment that is less conducive to cancer progression.

Curcumin induces epigenetic modifications that contribute to its antitumor effects. It inhibits the activity of DNA methyltransferases (DNMTs) and histone deacetylases (HDACs), leading to the reactivation of tumor suppressor genes and the repression of oncogenes [14,95,96]. Thus, curcumin influences gene expression in a manner favorable to fighting cancer.

Curcumin disrupts the β-catenin/TCF signaling pathway, which plays a crucial role in the development of various cancers, including **gastric and colorectal tumors**. It reduces the nuclear expression of β-catenin and the scaffold protein dishevelled, leading to the inhibition of β-catenin/TCF/LEF complex transactivation [97]. As a result, this downregulation suppresses tumor cell proliferation and promotes differentiation within neoplastic cells.

Although cancer cells rely primarily on anaerobic glycolysis (the Warburg effect), mitochondria play a key role in tumor proliferation, supporting both energy production and the processes required for rapid division. The reprogramming of mitochondrial function, including increased oxidative phosphorylation (OXPHOS) and mitochondrial biogenesis, contributes to chemotherapy resistance and metastatic potential. An important factor is the moderate production of reactive oxygen species (ROS), which activates signaling pathways involved in cell survival and proliferation (MAPK, PI3K/Akt, NF-κB). In this context, curcumin was able to directly inhibit the activity of mitochondrial ATP synthase (FoF1-ATP synthase) by binding to the F1 subunit. This inhibition reduces ATP synthesis and slows down the functioning of the electron transport chain, thus decreasing ROS production and blocking proliferative signaling associated with a pro-oxidative environment. Under conditions of mitochondrial damage, curcumin may, however, contribute to increased oxidative stress [66].

Curcumin and its analogs have demonstrated the ability to inhibit the Notch-1 signaling pathway, which is essential for tumor cell proliferation, especially in **pancreatic cancer**. This effect is achieved by reducing the expression of the Notch-1 receptor, its ligand, and the γ-secretase complex, responsible for activating Notch signaling. By blocking this pathway, curcumin disrupts the survival and self-renewal mechanisms of cancer cells, leading to the induction of apoptosis and inhibition of tumor growth [98].

The antitumor mechanisms described above—including the inhibition of cell proliferation, induction of apoptosis, suppression of oncogenic signaling pathways, and modulation of immune responses—have been demonstrated in preclinical studies across a wide range of malignancies, including colorectal, pancreatic, gastric, breast, prostate, brain, and neck cancers and non-small-cell lung cancer (NSCLC), as well as leukemia.

#### 2.2.3. Preclinical Applications and Clinical Trials

In a 4T1 triple-negative breast cancer murine model, treatment with curcumin and its derivative BHMC significantly reduced tumor burden, mitotic cells, lung metastases, and regenerative capacity [99]. Additionally, in a xenograft mouse model with Burkitt’s lymphoma, curcumin effectively inhibited tumor growth both in vivo and in vitro [100].

Regarding clinical trials, in a phase 1 clinical trial, 14 patients with advanced or metastatic breast cancer received a combination of docetaxel and curcumin. Patients received 100 mg/m^2^ of docetaxel as a one-hour intravenous infusion, and curcumin doses varied from 500 mg/day to dose-limiting toxicity. The combination of standard-dose docetaxel with 6 g/day of curcumin proved to be effective and safe against advanced breast cancer [101]. Another phase I/II clinical trial investigated the efficacy and safety of oral curcumin administration, alone or in combination with bioperine, in 29 patients with multiple myeloma (MM). Patients received escalating doses of curcumin (2, 4, 6, 8, or 12 g/day in two doses), alone or with 10 mg/day of bioperine, for 12 weeks. Based on the expression of biomarkers such as NF-κB (p65), COX-2, and phospho-STAT3, the combined treatment was more effective than bioperine alone, and curcumin was not associated with toxic effects [102].

In patients with advanced breast cancer, curcumin is currently being investigated as a monotherapy or in combination with paclitaxel [103]. The main objective of these clinical trials is to determine the effect of curcumin on the progression of advanced breast cancer and estimate the risk of adverse effects.

The use of curcumin in patients with localized, low-risk prostate cancer under active surveillance will be evaluated to assess its ability to slow tumor progression [104]. The potential radiosensitizing effect of curcumin in prostate cancer patients undergoing radiotherapy and its role as a radioprotector for normal tissues will also be analyzed [105].

In 2019, a combination treatment with curcumin and Avastin was administered to patients with unresectable metastatic colorectal cancer to evaluate progression-free survival and overall survival [106]. The efficacy and safety of curcumin in patients with advanced cervical cancer will be investigated in a phase 2 clinical trial, where treatment response rate will be the primary efficacy endpoint and CTCAE classification will be used to evaluate therapeutic safety [107].

A phase 2 clinical trial evaluated the tolerability of a combination of curcumin and cholecalciferol in the treatment of patients with untreated stage 0–II small lymphocytic lymphoma (SLL) or CLL. When administered together, the two substances suppressed cancer cell growth and increased the overall survival rate [108].

A study on curcumin, a naturally occurring triterpenoid, evaluated its ability to enhance the antitumor effect of celecoxib on MDA-MB-231 cells by downregulating COX-2 expression. This effect may be attributed to the induction of apoptosis. The research highlights the potential of the curcumin–celecoxib combination to increase the overall efficacy of chemotherapy while also reducing adverse effects [109].

### 2.3. Resveratrol

#### 2.3.1. Natural Sources, Chemical Properties, and Therapeutic Potential of Resveratrol

**Figure 3:**
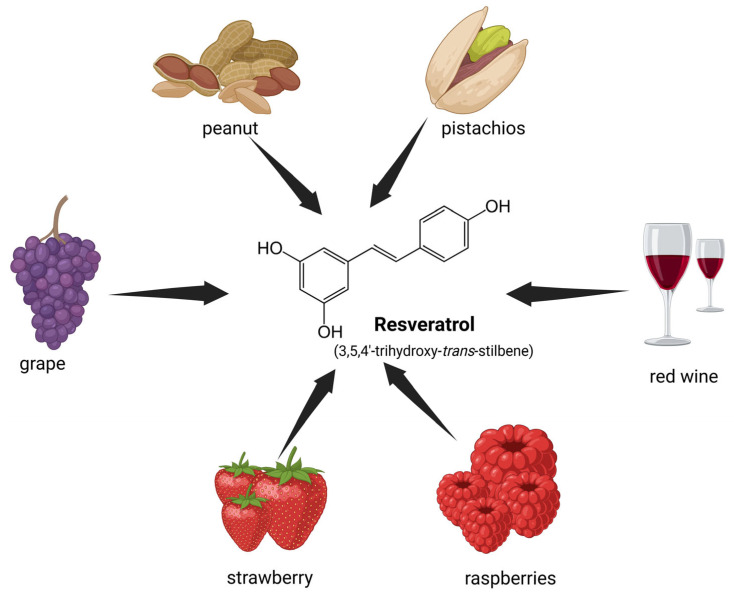
Natural sources of resveratrol.

Resveratrol (3,5,4′-trihydroxystilbene) is a natural, hydrophobic polyphenolic compound found in over 70 plant species, belonging to a wide range of botanical families including *Vitaceae*, *Fabaceae*, *Polygonaceae*, *Moraceae*, *Pinaceae*, *Gnetaceae*, *Liliaceae*, *Fagaceae*, *Myrtaceae*, *Cyperaceae*, and *Dipterocarpaceae* (Figure 3) [110]. The most important source of resveratrol is *Polygonum cuspidatum* (*Polygonaceae*), a plant used for centuries in traditional Asian medicine. The resveratrol content in *Polygonum cuspidatum* typically ranges from 3 mg to 6 mg per gram of dried rhizome [111,112]. The compound is also found in relatively high concentrations (50–100 μg/g) in the skin of red grapes (*Vitis vinifera*) and in varying concentrations in various berries, such as blueberries, cranberries, and mulberries [113,114]. Additionally, resveratrol can be found in peanuts and peanut-derived products, both raw and boiled, in variable amounts ranging from 0.3 to 15 μg/g and in in red wine in amounts ranging from 0.1 to 14.3 mg/L [115,116].

Resveratrol has two isomeric forms: trans-resveratrol and cis-resveratrol, with the trans isomer being considered more chemically stable and biologically active [117]. The compound is characterized by poor water solubility (~0.03 mg/mL), but it is soluble in ethanol and DMSO. In terms of stability, resveratrol is sensitive to light, heat, and oxidation, and UV radiation exposure induces the conversion of the trans form into the less stable cis form [118]. Its chemical structure is marked by the presence of three hydroxyl groups and a stilbene core, composed of two phenolic rings linked by a double bond. This architecture provides resveratrol with notable antioxidant properties, by enabling the donation of hydrogen atoms from the hydroxyl groups to neutralize free radicals [119,120]. Furthermore, the stilbene backbone facilitates interactions with various biomolecules and cellular receptors, which explains the diverse biological effects of the compound [120].

Resveratrol has attracted scientific interest due to its ability to delay or prevent the progression of several diseases, including cancer, cardiovascular diseases, and ischemic injury, as well as for its potential to enhance stress resistance and promote longevity in preclinical models [110,121]. This therapeutic versatility, consistently observed in in vivo studies, suggests that resveratrol may act through evolutionarily conserved mechanisms, including the activation of pathways similar to those triggered by caloric restriction—a process well known for its life-extending and disease-preventing effects [121,122].

Regarding its activity in cardiovascular diseases, resveratrol exhibits a multi-target pharmacological profile, validated in both preclinical and clinical studies. At the endothelial level, the compound stimulates nitric oxide (NO) production, a key mediator of vascular relaxation, contributing to improved vascular function and reduced blood pressure. Furthermore, the compound limits oxidative stress in myocardial tissue, inhibits platelet aggregation, and modulates lipid metabolism, thereby preventing the formation of atherosclerotic plaques. These mechanisms support the use of resveratrol as an adjuvant in cardiovascular disease prevention, particularly in the context of metabolic syndrome and chronic vascular inflammation [121,123,124].

Its anti-inflammatory activity is well documented and is based on the ability to inhibit proinflammatory signaling pathways. By blocking NF-κB activation, resveratrol downregulates genes involved in chronic inflammation, such as COX-2 and iNOS [125,126]. Additionally, it reduces circulating levels of proinflammatory cytokines (TNF-α, IL-1β, IL-6), thus helping to alleviate systemic inflammation [126,127,128]. Its efficacy has been demonstrated in models of arthritis, colitis, and inflammatory lung diseases, as well as in clinical trials showing significant reductions in inflammatory markers after supplementation with resveratrol natural extracts [121,129].

Resveratrol exhibits remarkable neuroprotective potential. This effect is largely due to its ability to cross the blood–brain barrier and modulate key mechanisms involved in neurodegeneration. It reduces oxidative stress in neural tissue, inhibits β-amyloid accumulation—a hallmark of Alzheimer’s disease—and activates SIRT1, a protein associated with neuronal survival and synaptic longevity [130,131]. Moreover, a recent study showed that resveratrol alleviates neuronal damage caused by cerebral ischemia–reperfusion injury. This neuroprotective action is achieved through the suppression of NR3C2 expression and inhibition of TRIM28, two molecular regulators involved in apoptosis and excessive autophagy [132]. Resveratrol administration led to a reduction in cerebral infarct volume, an improvement in cognitive performance, decreased levels of inflammatory cytokines, and the restoration of autophagy balance in both in vivo and in vitro models of cerebral ischemia–reperfusion injury [121,133,134].

Resveratrol exhibits significant anticancer potential, as demonstrated in numerous preclinical models [135]. Its mechanisms include inducing apoptosis in tumor cells, inhibiting cell proliferation and angiogenesis, and interfering with key oncogenic signaling pathways such as PI3K/Akt, MAPK, and Wnt [136]. Moreover, resveratrol may act synergistically with conventional cancer therapies, enhancing the sensitivity of tumor cells to chemotherapy or radiotherapy. While clinical evidence is still emerging, experimental findings provide a strong rationale for exploring resveratrol as an adjuvant in cancer therapy [137,138].

Finally, resveratrol is one of the few natural compounds shown to convincingly mimic the effects of caloric restriction, a mechanism well known for lifespan extension and metabolic protection. It activates SIRT1 and AMPK, enzymes involved in energy regulation, leading to improved insulin sensitivity, enhanced fatty acid oxidation, and reduced oxidative stress. Clinical studies in patients with type 2 diabetes have shown improvements in glycemic control and oxidative status, supporting its use in the prevention and treatment of metabolic syndrome [121,139].

#### 2.3.2. Antitumor Mechanisms of Resveratrol

Resveratrol limits the ability of tumor cells to multiply by blocking cell cycle progression, particularly at the G0/G1 phase. This effect is mediated by the downregulation of cyclins D1/D2 and CDKs, as well as the activation of the cyclin-dependent kinase inhibitor P21, which leads to the arrest of cell division [15,16]. Simultaneously, resveratrol induces apoptosis through the mitochondrial (intrinsic) pathway: it increases the expression of the pro-apoptotic protein Bax, decreases the level of the anti-apoptotic protein Bcl-2, and activates caspases 3 and 9, which are essential for the execution of programmed cell death [15,136]. The dual effect of resveratrol in inducing apoptosis and stimulating autophagy in cancer cells was investigated using chloroquine and small interfering RNAs targeting autophagy-related genes 5 and 7 to inhibit this process. The results suggested that RSV-induced autophagy might counteract its antitumor effects in Ishikawa cells, and combining it with chloroquine could represent a viable treatment option for certain **endometrial cancer** cells [140]. Another promising study identified 150 RSV target genes, 122 of which were associated with type I endometrial cancer. It highlighted key oncogenes and signaling pathways involved in the pharmacological effects of RSV on this cancer type [141].

Resveratrol inhibits the NF-κB signaling pathway, a major regulator of chronic inflammation and tumor cell survival. By blocking the nuclear translocation of the p65 subunit, it reduces the transcription of inflammatory genes such as COX-2, iNOS, and TNF-α. This effect directly influences the tumor microenvironment by decreasing the recruitment of pro-tumor cells and reducing tumor aggressiveness [16,17].

The effect of resveratrol on the cell viability and invasive potential of **gastric cancer** cells was analyzed using AGS and MKN45 cells treated with different doses of RSV (0–200 µM) for 24 h. The results showed that RSV increases SOD activity but reduces NF-kB transcriptional activity and the enzymatic activity of heparinase, correlating with a reduction in the invasive potential of gastric cancer cells [142].

Resveratrol interferes with several oncogenic signaling pathways, including PI3K/Akt, MAPK, and Wnt/β-catenin. Specifically, the inhibition of the PI3K/Akt pathway leads to decreased cell proliferation and activation of apoptotic mechanisms. Additionally, the activation of AMPK and inhibition of mTOR disrupt the abnormal metabolism of tumor cells and induce autophagy, a mechanism often associated with cell death in apoptosis-resistant cancers [16,143]. Transcriptomic analyses showed that LPA and RSV oppositely regulate genes associated with the epithelial–mesenchymal transition and autophagy, involving the PI3K-AKT, JAK-STAT, and Hedgehog pathways. LPA activates GLI1, BMI-1, SNAIL-1, and TWIST1 and inhibits autophagy, while RSV has the opposite effects. RSV counteracts LPA-induced malignancy by inhibiting the Hedgehog pathway and restoring autophagy, suggesting its inclusion in **ovarian cancer** therapy to reduce metastasis and chemotherapy resistance [144].. In vitro analyses confirmed the cytotoxic potential of RSV and pterostilbene, with low IC50 values and the ability to suppress the viral oncogene E6 [145].

Angiogenesis, a critical process for the nourishment and growth of solid tumors, is inhibited by resveratrol through downregulation of vascular endothelial growth factor (VEGF) and hypoxia-inducible factor 1-alpha (HIF-1α), a transcription factor involved in the cellular response to hypoxia. This effect reduces tumor vascular density and may significantly decrease tumor volume [16,146].

Resveratrol limits tumor cell invasion by inhibiting the proteolytic enzymes MMP-2 and MMP-9, which degrade the extracellular matrix and facilitate cancer cell migration. It also increases the expression of E-cadherin, a key cell adhesion molecule, contributing to the maintenance of the epithelial phenotype and suppression of the epithelial–mesenchymal transition (EMT) [16,136].

Due to its phenolic structure, resveratrol acts as a potent antioxidant by neutralizing reactive oxygen species (ROS) and preventing oxidative damage to DNA, proteins, and cellular lipids. This protective effect plays a crucial role in preventing mutations and oxidative stress associated with cancer initiation and progression [136,146].

Resveratrol influences the expression of cancer-related genes through epigenetic modulation. It regulates microRNAs (e.g., miR-34a, miR-21) and modifies histone acetylation, contributing to the activation of tumor suppressor genes and repression of oncogenes. These changes help control proliferation and increase tumor cell sensitivity to treatment [15].

Resveratrol enhances the chemotherapeutic effect of agents such as 5-fluorouracil (5-FU), cisplatin, and doxorubicin by inhibiting the expression of MDR1 (P-glycoprotein), a drug efflux pump associated with multidrug resistance. It also interferes with signaling pathways involved in resistance, thereby improving the efficacy of anticancer therapy [16,136].

The antitumor mechanisms described above—including the inhibition of cell proliferation, induction of apoptosis, suppression of oncogenic signaling pathways, and modulation of the tumor microenvironment—have been demonstrated in preclinical studies across a broad spectrum of malignancies. These include colorectal cancer, breast cancer, prostate cancer, non-small-cell lung cancer (NSCLC), pancreatic cancer, skin cancer (including melanoma), ovarian cancer, hematologic malignancies such as leukemia and multiple myeloma, and brain tumors such as glioblastoma [136,137].

#### 2.3.3. Preclinical Applications and Clinical Trials

The antitumor effect of resveratrol-loaded topical invasomes was evaluated in vivo in Ehrlich-tumor-bearing mouse models. The results showed that the group treated with a resveratrol invasomal gel had the smallest tumor volume, with no signs of organ toxicity, indicating the resveratrol’s safety in **skin cancer** treatment. Invasomes are considered promising lipid nanoscale systems for topical resveratrol delivery, with high skin penetration ability and anticancer effects in **cutaneous carcinoma** treatment. Increased expression of BAX and caspase-3 genes and decreased protein levels of NF-kB and BCL2 were demonstrated via RT-PCR and ELISA tests [147].

Researchers investigated the effects of resveratrol administration on Ki-67 proliferation marker expression in **colorectal** tissue. Resveratrol was administered in doses of 0.5 or 1 g/day for eight days, and the results showed a significant 5% decrease in tumor cell proliferation, indicating that resveratrol inhibits cellular proliferation in colorectal tissue [148].

In vivo studies on mice with TC1 tumors showed that resveratrol and pterostilbene significantly inhibited tumor development. Pterostilbene induced tumor cell apoptosis (via caspase-3 activation), while resveratrol caused cell cycle arrest (by decreasing PCNA expression) [145]. Another HPV-induced cancer study showed that both compounds reduced the oncogenic protein E6, activated caspase-3, and increased p53 protein expression, with pterostilbene being more effective [149].

In xenograft models, RSV delayed tumor growth and increased survival rates, especially in RKO tumors with high expression of the G-protein-coupled estrogen receptor (GPER). Resveratrol significantly inhibited the viability of GPER-high-expressing organoids [150].

In another study on patients with **colorectal cancer** and liver metastases, researchers examined the effects of the micronized resveratrol formulation SRT501 on liver tissue. Patients received supplementation with SRT501 at a dose of 5 g/day for two weeks. The study observed an increase in cleaved caspase-3 levels in the liver tissue of patients who received SRT501. Cleaved caspase-3 is a marker of apoptosis, suggesting increased apoptotic activity in cancerous tissue compared to the placebo group [151].

The first clinical study investigating resveratrol in the context of cancer analyzed the effects of freeze-dried grape powder (GP), which contains resveratrol and plant-derived resveratrol compounds, on the Wnt signaling pathway, known for its involvement in colorectal carcinogenesis, in both colon cancer and normal colonic mucosa [152]. It was observed that GP administration (80 g/day, containing 0.07 mg of resveratrol) for two weeks led to reduced expression of Wnt target genes in normal colonic mucosa but had no effect on cancerous mucosa. This suggests that GP or resveratrol may have a preventive rather than therapeutic role in colon cancer.

The effects of administering resveratrol in doses of 0.5 or 1 g/day for eight days on Ki-67 proliferation marker expression in colorectal tissue have been studied, and a 5% reduction in tumor cell proliferation was reported [148].

In patients with colorectal cancer and liver metastases, supplementation with SRT501 (a micronized resveratrol formulation produced by Sirtris Pharmaceuticals, a GSK company, Cambridge, MA, USA) at a dose of 5 g/day for two weeks increased cleaved caspase-3 levels in liver tissue, suggesting increased apoptosis in cancerous tissue compared to subjects treated with a placebo [151].

In a phase 1 trial on muscadine grape skin extract, patients with biochemically recurrent **prostate cancer** were administered a high dose (4000 mg/patient) of powdered muscadine grape skin (Vitis rotundifolia), which contains ellagic acid, quercetin, and resveratrol. The treatment was found to be safe and warrants further investigation in a phase II dose-evaluation trial [153].

In another randomized, placebo-controlled clinical trial using two doses of resveratrol (150 mg or 1000 mg/day) over four months, a significant reduction in serum levels of androstenedione, dehydroepiandrosterone, and dehydroepiandrosterone sulfate was observed, while prostate size remained unaffected in patients with benign prostatic hyperplasia [154].

In addition to effects in cancer patients, resveratrol has also shown effects in individuals at high risk for cancer. For example, supplementation with 50 mg resveratrol twice daily for 12 weeks reduced DNA methylation of the tumor suppressor gene Ras association domain-containing protein 1 (RASSF1A) in the breast tissue of women at high risk for **breast cancer** [155].

Resveratrol supplementation has also been shown to have beneficial effects on estrogen metabolism, thus reducing **breast cancer** risk factors in overweight and obese postmenopausal women [156].

A study conducted on in vitro experiments using human **liver cancer** HepG2 cells and in vivo experiments using H22 tumor-bearing mice utilized resveratrol (RV) loaded on HAS (human serum albumin) nanoparticles in combination with folic acid. It was observed that this formulation resulted in a slower drug release at the injection site. On the other hand, RV encapsulated in HSA without folic acid led to significant resveratrol accumulation in the tumor. The study showed that the bioavailability of RV was significantly increased when loaded on HSA nanoparticles in combination with folic acid. The bioavailability of RV in this form was six times higher than free RV without nanoparticle encapsulation [157].

In a study involving liposomes synthesized and loaded with resveratrol (RV) and paclitaxel, researchers investigated their ability to overcome drug resistance in **breast cancer** cells. The study’s main findings revealed that the liposomes exhibited significant cytotoxic effects on drug-resistant MCF-7/ADR tumor cells. Moreover, they demonstrated improved bioavailability and enhanced tumor retention in mice bearing drug-resistant tumors [158].

## 3. Pharmacokinetic and Biochemical Limitations of Natural Compounds

Although the three plant-derived molecules discussed, rhein, curcumin, and resveratrol, have shown promising potential in combating cancer and its related complications in preclinical studies, their clinical application is significantly limited by low systemic bioavailability. This limitation is caused by a number of factors, including inadequate molecular size, low solubility in water, high lipophilicity, rapid metabolism, limited cellular uptake, and their complex interaction with P-glycoprotein (P-gp), acting both as substrates and modulators of its efflux activity [159,160,161,162,163].

Rhein, a natural anthraquinone with recognized antitumor potential, is limited in its clinical applicability due to several major pharmacokinetic barriers. When administered orally in its conventional form, rhein exhibits poor systemic absorption, a short half-life, and rapid elimination, resulting in subtherapeutic exposure at target sites. These limitations are mostly due to its low aqueous solubility, which prevents effective dissolution in the gastrointestinal tract, an essential step in optimal oral absorption. Its hydrophobic nature exacerbates this problem, resulting in poor intestinal absorption and, subsequently, reduced systemic bioavailability. In addition, like other anthraquinone derivatives, rhein undergoes extensive hepatic metabolism, which further reduces the concentration of the active compound in the systemic circulation.

Preclinical pharmacokinetic studies in rats have reported an area under the curve (AUC) of only 7.32 µg/h/mL and a peak plasma concentration (Cmax) of 1.96 µg/mL, values that reflect extremely limited in vivo availability [164]. Complementing these findings, clinical studies in healthy volunteers have shown that after oral administration of *Rheum Undulatum* L. extract, rhein was the only detectable compound in plasma, with a mean elimination half-life of 3.39 ± 0.35 h, indicating rapid systemic clearance [165].

A study by Teng et al., published in 2022, revealed that rhein is capable of inhibiting P-glycoprotein (P-gp) through two complementary mechanisms [162]. On one hand, rhein acts as a noncompetitive inhibitor of P-gp’s efflux activity, and on the other hand, it also reduces the expression of P-gp by interfering with the activation of STAT3, a signaling pathway known to regulate multidrug resistance [161,162]. These dual actions suggest that rhein may contribute both to enhancing the intracellular retention of chemotherapeutic agents and to attenuating mechanisms that promote drug efflux in tumor cells. As a result of these dual actions, rhein significantly increases the intracellular accumulation of co-administered drugs and shows a very strong synergistic effect with vincristine, with a reported combination index (CI) of 0.092, indicating high synergism. An important advantage is that rhein can actually boost its own effectiveness. By blocking the cellular “pumps” (P-gp) that normally push drugs out of cancer cells, rhein essentially creates a feedback loop where it helps more of itself accumulate inside tumor cells. This means it naturally increases its concentration exactly where it needs to work. This self-reinforcing property makes rhein especially promising for targeted cancer treatments and for fighting cancers that have become resistant to multiple drugs [162].

Even in situations where this advantage of rhein could be exploited, all the other pharmacokinetic limitations of the compound make it difficult to use in cancer prevention or treatment in its natural form. Therefore, advanced formulations are needed, aiming to improve rhein’s solubility, chemical stability, and intestinal absorption, ultimately enhancing its clinical performance in oncology.

Curcumin is well known for its anti-inflammatory and antitumor properties, manifested by the inhibition of signaling pathways such as NF-κB, STAT3, and VEGF [166,167]. However, its clinical applicability in oncology is deeply limited by an unfavorable pharmacokinetic profile.

One of the most significant disadvantages of curcumin is its extremely low oral bioavailability, with less than 1% of the supplied amount entering the systemic circulation. The molecule’s low water solubility (~8 mg/L) and poor intestinal absorption make it difficult to permeate the gastrointestinal barrier effectively [168,169]. After absorption, curcumin is quickly metabolized in the intestines and liver by glucuronidation and sulfation. These processes lead to the formation of inactive metabolites, including curcumin glucuronide, curcumin sulfate, tetrahydro curcumin, and hexahydro curcumin [168,170]. On top of that, curcumin does not persist in the body for a long period of time; it is cleared from the bloodstream in less than 2 h and mostly passes out through feces.

Addressing drug resistance, curcumin targets P-gp, a key protein that acts like a cellular security guard pushing drugs out of cells. To do that, curcumin works in two ways: it directly interferes with this pump and also disrupts the internal cellular communication pathways (like PI3K/Akt/NF-κB) that control it. By blocking P-gp, curcumin helps itself and other cancer drugs build up inside cancer cells rather than being ejected. This effect is especially important in the digestive tract, where these pumps are found in high numbers and often prevent treatments from working effectively. Although curcumin has low systemic bioavailability due to the aspects presented above, its ability to inhibit P-gp may serve as a local self-potentiating mechanism. In regions where curcumin could be directly applied or selectively delivered, blocking P-gp-mediated transport can enhance its absorption and help maintain higher intracellular levels. However, this mechanism alone cannot compensate for curcumin’s general pharmacokinetic limitations.

Although curcumin has excellent anticancer properties, systemic treatment efficiency cannot be obtained without the use of specialized formulations such as nanoemulsions, liposomes, or polymeric delivery vehicles, which improve its solubility, metabolic protection, and absorption.

Resveratrol is a natural polyphenolic compound with demonstrated anticancer activity through multiple mechanisms, including the induction of apoptosis, regulation of the cell cycle, inhibition of inflammation, limitation of angiogenesis, prevention of metastasis, modulation of gene expression, targeting of cancer stem cells, influence on the tumor microenvironment, enhancement of chemotherapeutic efficacy, and modulation of redox and hormone signaling [171]. However, its clinical use is significantly hampered by several unfavorable pharmacokinetic characteristics.

Despite its promising pharmacodynamic profile, the clinical translation of resveratrol remains limited due to several pharmacokinetic drawbacks. One of the main challenges is its very low water solubility (~30 mg/L), which significantly hinders its dissolution and gastrointestinal absorption [172]. Furthermore, once absorbed, resveratrol undergoes extensive metabolism in the intestine and liver, primarily forming glucuronide and sulfate conjugates, which are biologically inactive. As a result, systemic bioavailability is estimated to be only 1–5%, with plasma concentrations consisting almost entirely of these metabolites rather than the free, active form [172].

Another important limitation is its instability, particularly the isomerization of trans-resveratrol into the less active cis form when exposed to light or unfavorable environmental conditions [173]. Additionally, the compound shows limited distribution to tumor tissues, especially in the absence of targeted delivery strategies.

Although improving resveratrol’s bioavailability through P-glycoprotein (P-gp) inhibition might appear to be a plausible strategy, current evidence suggests otherwise. A study conducted by Junco et al. (2013) demonstrated that while resveratrol can sensitize cancer cells to chemotherapeutic agents such as ursolic acid, it does not inhibit the efflux of Rhodamine 123, a known P-gp substrate, which indicates that it does not directly inhibit P-gp [163]. However, other studies have reported that resveratrol may act as an indirect modulator of P-gp expression, potentially by interfering with intracellular signaling pathways such as NF-κB [174].

Consequently, while resveratrol does not significantly enhance its own bioavailability through direct P-gp inhibition, it may play a valuable role in potentiating the effects of other anticancer agents. Indirectly, it may also contribute to a reduction in P-gp expression within tumor tissues, which could be beneficial in overcoming multidrug resistance.

To address its major pharmacokinetic limitations, low solubility, rapid metabolism, and poor tissue distribution, numerous nanotechnology-based formulations have been developed. These include lipid-based nanoparticles, polymeric delivery systems, and other advanced carriers designed to enhance solubility, chemical stability, absorption, and targeted delivery, thereby maximizing the therapeutic potential of resveratrol in clinical settings [160].

## 4. Nanotechnology as a Strategy for Enhancing Antitumor Efficacy

### 4.1. Introduction to Nanotechnology-Based Drug Delivery Systems in Cancer Therapy

Nanotechnology has developed as a transformative approach in order to improve drug delivery in the human body, offering the possibility to create targeted delivery systems to enhance drug efficacy while reducing adverse effects. Nanomedicine, the application of nanotechnology in healthcare, uses materials and devices designed at the nanoscale dimensions (1–100 nm) to interact with biological systems at the molecular level [175]. These nanoscale dimensions are very useful in medicine because they match the size range of most biological molecules and structures, allowing for novel interactions with cellular components. Nanoparticles employed in drug delivery possess unique physicochemical properties such as high surface-to-volume ratio, adjustable size, shape, and surface characteristics, which can be properly designed to optimize drug bioavailability, stability, and accumulation at target sites [176]. The main principle behind nanotechnology-based drug delivery systems is to overcome biological barriers that traditionally limit therapeutic efficacy. These barriers include enzymatic degradation, poor water solubility, fast clearance by the reticuloendothelial system (RES), and limited ability to cross biological membranes such as the blood–brain barrier [177].

Cancer therapy represents one of the most prominent applications of nanotechnology in medicine, where conventional treatments often suffer from significant limitations including systemic toxicity, poor tumor selectivity, and development of drug resistance. Nanomaterials offer multiple advantages in addressing these challenges, thereby enhancing therapeutic efficacy while minimizing adverse effects.

The evolution of nanomedicine in cancer therapy has been marked by successive generations of development. First-generation nanoparticles in oncology focused primarily on passive targeting via the enhanced permeability and retention (EPR) effect, wherein nanoparticles accumulate preferentially in tumor tissues due to their leaky vasculature and impaired lymphatic drainage [178]. Second-generation nanoparticles incorporated active targeting strategies through surface functionalization with ligands that recognize specific receptors overexpressed on target cells. The current third-generation nanotechnology platforms integrate stimuli-responsive elements that enable the controlled release of therapeutic agents in response to specific environmental triggers such as pH, temperature, enzymes, or externally applied stimuli like light or ultrasound [179].

The EPR effect plays an essential role in the application of nanomedicine in cancer treatment. Solid tumors often have abnormal vascularization characterized by dispersed architecture, increased permeability, and impaired lymphatic drainage. Nanoparticles of suitable dimensions (often 30–200 nm) can passively aggregate in tumor tissues via permeable blood arteries and remain trapped due to compromised lymphatic clearance [180]. Preclinical investigations have shown that nanoformulations can achieve drug concentrations in tumor tissue that are 1.5 to 5 times higher compared to free drug formulations, highlighting the crucial role of the EPR effect in enhancing treatment efficacy [181].

Beyond passive targeting via the EPR effect, nanoparticles can be engineered with surface ligands that selectively bind to receptors overexpressed on cancer cells. Antibodies, peptides, aptamers, and small compounds that recognize specific tumor markers like folate receptors, transferrin receptors, or epidermal growth factor receptors are common targeted ligands [182]. This active targeting approach enhances cellular internalization and retention of therapeutic agents within cancer cells. Evidence from studies suggests that actively targeted nanoparticles can attain 3–10 times greater cellular absorption than non-targeted alternatives, significantly improving therapeutic efficacy [183].

Multidrug resistance (MDR) remains a major obstacle in cancer therapy, often mediated by ATP-binding cassette transporters that actively efflux drugs from cancer cells. Nanoparticles can overcome MDR through multiple mechanisms: by bypassing membrane-bound efflux pumps through endocytosis, co-delivering MDR inhibitors with chemotherapeutic agents, or depleting ATP required for efflux pump activity [184]. Clinical studies have shown that nanoformulations exhibit improved efficacy in MDR tumors, with response rates 15–30% higher than those achieved with conventional formulations [185].

Nanoparticles provide an excellent platform for integrating multiple therapeutic modalities within a single delivery system. These nanoplatforms can integrate imaging agents with therapeutic agents, enabling real-time monitoring of drug delivery and treatment response. Furthermore, nanoparticles can be designed to incorporate combinations of chemotherapeutic agents, photosensitizers for photodynamic therapy, genetic material for gene therapy, or immunomodulatory agents for immunotherapy [186]. A recent study revealed that multimodal nanotherapeutics, integrating chemotherapy and immunotherapy, led to complete tumor regression in 87% of treated mice, in contrast to 0% with chemotherapy alone and 15% with immunotherapy alone [187].

Nanoencapsulation of chemotherapeutics significantly modifies their pharmacokinetic profile, basically resulting in extended half-lives and modified biodistribution patterns. PEGylated liposomal doxorubicin (Doxil^®^/Caelyx^®^) exhibits a plasma half-life of approximately 55 h, in contrast to the ten minutes associated with free doxorubicin, thereby enhancing tumor exposure to the chemotherapeutic agent [188]. Furthermore, nanoformulations can prevent off-target toxicity by reducing the medication exposure of healthy tissues. Clinical trials indicate that liposomal doxorubicin results in an 80% reduction in cardiotoxicity relative to conventional doxorubicin, while preserving similar or enhanced antitumor efficacy [189].

Nanomedicine includes several nanocarrier systems, each holding unique structural features, synthesis techniques, and therapeutic uses. Understanding these various nanoplatforms is important for rational design and selection of suitable delivery methods for specific therapeutic goals.

Liposomes are spherical vesicles that contain phospholipid bilayers coating an aqueous nucleus. Their amphiphilic structure enables the encapsulation of both hydrophilic substances (in the aqueous nucleus) and hydrophobic agents (inside the lipid bilayer). Since Doxil^®^ became the first FDA-approved liposomal chemotherapeutic drug in 1995, numerous liposomal formulations have entered clinical use, including AmBisome^®^ (amphotericin B), Marqibo^®^ (vincristine), and Onivyde^®^ (irinotecan) [190]. Research has shown that liposomal drug delivery can improve the therapeutic index by three to five times compared to free drug formulations in multiple clinical applications [191].

Lipid nanoemulsions are heterogeneous colloidal systems, formed of very fine droplets (with dimensions between 10 and 200 nm) of a lipid phase (internal phase) dispersed in an external aqueous phase, stabilized using surfactants and co-surfactants [192]. The stability of these nanoemulsions is particularly important for pharmaceutical products and depends greatly on the ratio between the lipid phase and surfactant, the physicochemical properties of the components, and the preparation method used [192,193]. Lipid nanoemulsions are frequently used for active substance delivery due to their capacity to load both hydrophilic and hydrophobic compounds. They are also used for photosensitive substances, being the most suitable systems for photodynamic and photothermal therapy. These nanoemulsions can be loaded with photosensitizing substances, ensuring their stability in systemic circulation and allowing targeted activation only in the presence of specific light radiation. Thus, systemic toxicity is significantly reduced, and therapeutic efficiency is significantly improved, especially in antitumor treatments [194]. Due to their high loading capacity and relatively easily controllable dimensions, these structures are also suitable for diagnostic applications, especially as imaging contrast agents. They can incorporate fluorescent molecules, radioisotopes, or MRI imaging agents, helping with the precise and early diagnosis of various pathologies [195]. Lipid nanoemulsions are a promising and versatile platform in the field of pharmaceutical and biomedical products, offering multiple advantages regarding stability, release control, biodistribution, and reduced toxicity.

Solid lipid nanoparticles (SLNs) consist of a solid lipid core stabilized by surfactants, while nanostructured lipid carriers (NLCs) incorporate liquid lipids into the solid matrix to enhance drug loading capacity and stability. These systems offer advantages including ease of large-scale production, absence of organic solvents in preparation, and enhanced stability compared to liposomes [196]. Evidence from studies has indicated that SLNs and NLCs can enhance the oral bioavailability of poorly water-soluble drugs by 2- to 3-fold compared to traditional formulations [197].

Polymeric micelles are self-assembling colloidal systems composed of amphiphilic block copolymers. These nanostructures typically possess a hydrophobic core that encapsulates poorly water-soluble drugs and a hydrophilic shell, a structure that provides stability in aqueous environments. With sizes typically ranging from 10 to 100 nm, polymeric micelles are particularly effective at penetrating solid tumors and avoiding rapid renal clearance [198]. Several micellar formulations have advanced to late-stage clinical trials, including Genexol-PM^®^ (paclitaxel-loaded PEG-PLA micelles) and NK012 (SN-38-loaded PEG-PGA micelles). Clinical studies have shown that polymeric micellar formulations can reduce adverse effects by 30–50% while maintaining or improving efficacy compared to conventional formulations [199].

Dendrimers are highly branched, monodisperse macromolecules with a well-defined tree-like architecture [200]. Their unique structure includes an initiator core, branching units, and multivalent surface groups that can be functionalized for targeted delivery or improved biocompatibility. The precisely controlled structure of dendrimers enables exceptional control over size, molecular weight, and surface properties, making them valuable for applications requiring high reproducibility [201]. Scientific research has shown that dendrimers are capable of enhancing the solubility of poorly water-soluble drugs by up to 10,000-fold and improving transepithelial transport by 3- to 15-fold relative to free drug formulations [202].

Metal-based nanoparticles including gold, silver, and iron oxide have attracted significant attention due to their unique optical, magnetic, and electronic properties. Gold nanoparticles are particularly valuable for photothermal therapy due to their surface plasmon resonance, while superparamagnetic iron oxide nanoparticles (SPIONs) serve as contrast agents for magnetic resonance imaging and magnetothermal therapy [203]. Clinical studies with SPIONs in magnetic hyperthermia treatment for glioblastoma demonstrated a median overall survival of 23.9 months compared to 14.6 months with standard therapy alone [204].

Mesoporous silica nanoparticles (MSNs) have a honeycomb-like structure with many pores, providing a high surface area (>700 m^2^/g) and pore volume for drug loading. The silica framework offers great biocompatibility, stability, and ease of surface functionalization. MSNs are particularly valuable for controlled and sustained drug release applications and can be designed with stimuli-responsive gatekeepers that regulate payload release in response to specific triggers [205]. According to study results, MSNs can achieve sustained drug release profiles extending over periods from hours to weeks, with 2–5 fold higher drug loading capacity compared to other polymeric systems [206].

Recognizing that no single nanocarrier system possesses all desired characteristics for effective drug delivery, researchers have developed various hybrid nanostructures that combine the advantages of different materials. Examples include lipid–polymer hybrid nanoparticles, which integrate the biocompatibility of lipids with the structural integrity of polymers, and organic–inorganic hybrid nanocomposites that combine the versatility of organic materials with the unique properties of inorganic components [207]. A comparative analysis of hybrid lipid–polymer nanoparticles with their individual components demonstrated 27% higher cellular uptake, 30% lower cytotoxicity, and 34% greater therapeutic efficacy in preclinical models [208].

While significant progress has been made in translating nanotherapeutics from bench to bedside, with dozens of FDA-approved nanomedicines currently in clinical use, numerous challenges remain. These include scaling up manufacturing processes while maintaining batch-to-batch consistency, addressing potential long-term toxicity concerns, and overcoming biological barriers to improve delivery efficiency. Nevertheless, ongoing advances in materials science, bioengineering, and pharmaceutical technology continue to expand the frontiers of nanomedicine, promising increasingly sophisticated and effective therapeutic strategies in the future.

### 4.2. Nanocarrier Systems for Rhein, Curcumin, and Resveratrol in Cancer Therapy

Natural compounds such as rhein, curcumin, and resveratrol represent a promising class of anticancer agents due to their ability to modulate multiple signaling pathways, induce apoptosis, inhibit angiogenesis, and suppress tumor growth. Despite their impressive therapeutic potential demonstrated in preclinical studies, clinical translation has been severely limited by their poor aqueous solubility, rapid metabolism, low bioavailability, and insufficient tumor accumulation [143,209,210]. Despite resveratrol being available in traditional dosage forms like tablets and capsules, there is insufficient evidence regarding its efficacy against cancer, primarily due to these pharmacokinetic limitations [143,211].

Nanotechnology has emerged as a promising solution to overcome these biopharmaceutical challenges. Through precise engineering of various nanocarrier systems—including liposomes, polymeric nanoparticles, lipid nanoemulsions, solid lipid nanoparticles, and polymeric nanoparticles—researchers have successfully enhanced the pharmacokinetic profiles, stability, tumor-targeting capabilities, and therapeutic efficacy of these compounds [212,213]. A key benefit of nanocarriers is their ability to efficiently permeate tumor tissues through the enhanced permeability and retention (EPR) effect while being prevented from entering normal tissues by intact blood vessels [213]. This targeted delivery mechanism increases the concentration of therapeutic agents at tumor sites while reducing their distribution in healthy tissues, thereby minimizing potential toxicity [143,213].

For resveratrol specifically, various nanocarriers have been developed, including lipid nanocarriers, liposomes, polymeric nanoparticles, and solid dispersions that facilitate rapid absorption and increase plasma concentrations [173,212]. These nanoformulations protect resveratrol from degradation, enhance stability and solubility, control delivery, and exhibit selective cytotoxicity limited to cancer cells [214]. Similarly, nanotechnology approaches have improved the efficacy of curcumin by enhancing its aqueous solubility and targeted delivery [215,216]. Polymer nanoparticles are particularly valuable due to their high encapsulation efficiency, which reduces the required amount of carrier material and minimizes potential toxic effects [217]. Furthermore, nanocarriers can be customized with targeting moieties such as antibodies or peptides to enhance their specificity for diseased tissues or cells [218]. This customization enables higher concentrations of therapeutic compounds at the target site, leading to improved therapeutic outcomes while potentially reducing side effects and overcoming resistance to therapy [210,218].

#### 4.2.1. Liposomes

Liposomes represent one of the most promising nanocarrier systems for delivering poorly water-soluble compounds like rhein, resveratrol, and curcumin to tumor sites. Research has demonstrated that encapsulation in liposomes significantly enhances the biological activity and antioxidant effects of resveratrol compared to its free form [219]. These self-assembled structures feature a hydrophilic polymeric shell and a hydrophobic core, which allows for efficient loading of both water-soluble and lipophilic compounds while providing enhanced permeability at tumor sites [220].

A major advantage of liposomal formulations is their ability to protect encapsulated compounds from degradation while achieving targeted delivery. For example, liposomal rhein showed cytotoxic effects against MCF-7 tumor cells at dosages reduced by 2.5 times compared to free rhein, while maintaining low cytotoxicity on normal cells [26]. The co-encapsulation of multiple compounds in liposomes offers additional advantages through synergistic effects. Huang and colleagues demonstrated that co-loading curcumin and resveratrol in liposomes achieved optimal encapsulation rates and antioxidant activity when the compounds were mixed at a 5:1 ratio (curcumin/resveratrol) [221].

Despite their advantages, conventional liposomes face limitations, including a short half-life and susceptibility to oxidation and hydrolysis. Intravenous administration of resveratrol typically results in a short half-life of only 7.8 to 33 min [219]. To address these challenges, researchers have developed modified liposomal systems. PEGylation—the grafting of polyethylene glycol chains onto liposomes—has been employed to increase half-life and extend blood circulation time for resveratrol delivery [219]. Another notable example is PEGylated liposomes co-encapsulating paclitaxel and resveratrol, which enhanced bioavailability, tumor accumulation, and cytotoxicity in drug-resistant breast cancer cells, resulting in significant tumor growth inhibition without additional systemic toxicity [158].

Liposomal curcumin (~100 nm) has shown remarkable antitumor activity when administered systemically in colorectal cancer models. In preclinical studies with LoVo and Colo205 cell lines, liposomal curcumin effectively inhibited tumor growth and induced apoptosis both in vitro and in vivo. Notably, it demonstrated synergistic effects when combined with oxaliplatin at a 4:1 ratio in LoVo cells, and in Colo205 xenografts, it outperformed oxaliplatin while reducing the expression of key cancer markers including VEGF, IL-8, and CD31 [222].

Advanced formulations continue to emerge, addressing specific delivery challenges. For example, curcumin-loaded CaCO_3_-encapsulated liposomes (LCCs) with pH sensitivity have been developed to enhance cytosolic delivery and lysosomal escape in colorectal cancer. These smart delivery systems showed superior antitumor efficacy compared to standard liposomes in an AOM/DSS-induced murine model, attributed to their ability to rapidly release their cargo in acidic lysosomal environments while maintaining prolonged circulation in the bloodstream [223].

Rhein-loaded liposomal formulations have also shown promising clinical potential. Rhein-loaded hyaluronic acid-modified cationic liposomes (HA-Lip-rhein, ~190 nm) demonstrated enhanced cellular uptake and cytotoxicity in 4T1 breast cancer cells, with significant anti-metastatic activity. In vivo studies confirmed these formulations effectively reduced lung metastases and exhibited superior tumor targeting compared to both free rhein and unmodified liposomes, indicating their readiness for further clinical development [11].

Commercial applications of liposomal delivery systems are already emerging, as seen with curcumin/piperine-loaded liposomes (Meriva products), which demonstrated significantly enhanced anti-inflammatory properties compared to unformulated curcuminoids in clinical trials [11,224,225]. These formulations show improved resistance to acidic stomach pH and better intestinal absorption through biliary emulsions [224].

#### 4.2.2. Nanoemulsions

Innovative nanoemulsions are also under development to optimize the resistance and biodistribution of natural compounds.

Curcumin nanoemulsions have demonstrated impressive anticancer activities across various cancer types. For example, curcumin nanoemulsions (44 nm) formulated with modified lecithin significantly reduced tumorigenesis (91.81% decrease) and tumor area (89.95% reduction) in transgenic mice while preventing the development of microinvasive squamous cell carcinoma [226]. Studies on breast cancer cell lines showed that gelatine-encapsulated curcumin nanoemulsions significantly stabilized curcumin and increased its bioavailability compared to the free compound [227]. Similarly, curcumin-loaded nanoemulsion formulations exhibited high phototoxic effects, minimizing cell proliferation and enhancing reactive oxygen species generation in human breast adenocarcinoma cells [227]. The anticancer effects of nanoemulsions extend to other cancer types as well. In prostate cancer, curcumin-loaded nanoemulsions showed higher cytotoxicity than free curcumin [228].

Combination approaches using nanoemulsions have shown particularly promising results. A self-microemulsifying formulation containing both curcumin and resveratrol (15–20 nm droplets) demonstrated synergistic antioxidant activity and enhanced cytotoxicity against HT-29 colon cancer cells (IC50 = 18.25 μM) while dramatically improving oral bioavailability for both compounds. Similarly, curcumin/5-fluorouracil co-loaded agarose–chitosan nanoemulsions showed high loading efficiency (85.6%) and entrapment efficiency (79.2%) for curcumin, with pH-sensitive release characteristics that enhanced cytotoxicity against MCF-7 breast cancer cells [229].

Advanced nanoemulsion formulations can respond to specific stimuli in the tumor microenvironment (TME). For instance, GSH-sensitive nanomicelles loaded with curcumin have been designed to respond to GSH in the TME, improving delivery efficacy to tumor sites [230,231]. In vivo pharmacokinetic research showed that loading curcumin into nanomicelles significantly improved its plasma concentration, bioavailability, and half-life [230,231].

Combination therapies with nanoemulsions can provide synergistic effects through complementary mechanisms. For example, a tumor-targeted nanomicelle loaded with resveratrol and the photodynamic reagent Ce6 was developed to treat oral squamous cell carcinoma by simultaneously triggering the autophagic cell death and apoptosis of cancer cells [230,232]. This approach enhanced photodynamic therapy (PDT) by allowing resveratrol to regulate hypoxia, providing sufficient oxygen for PDT, which consequently activated PDT to produce reactive oxygen species (ROS) [230,232].

Curcumin has been demonstrated to increase the responsiveness of cancer cells to chemotherapy, making it a beneficial component in these formulations [233,234]. These natural compounds can also help mitigate the side effects of conventional chemotherapy. For instance, resveratrol has been combined with doxorubicin in liposomal formulations to reduce cardiotoxicity while maintaining or enhancing anticancer efficacy [233].

Overall, nanoemulsion formulations have emerged as optimal carriers for bioactive compounds like curcumin, resveratrol, and quercetin, enabling them to overcome physiological barriers, avoid drug metabolism, and achieve delivery at higher concentrations at cancer sites [212]. The enhanced anticancer efficacy, increased tumor cell targeting, and reduced toxicity of these formulations have positioned nanoemulsions as a promising platform for improving the therapeutic potential of these natural compounds [212].

#### 4.2.3. Solid Lipid Nanoparticles (SLNs)

Solid lipid nanoparticles (SLNs) represent an important class of lipid-based delivery systems for natural compounds with anticancer properties. These nanocarriers are composed of solid lipids at body temperature and offer advantages including high drug loading capacity, protection from degradation, and controlled-release properties. For curcumin delivery, SLNs have shown significant potential in enhancing anticancer activity. Curcumin-loaded solid lipid nanoparticles (311 nm) synthesized via solvent microemulsification demonstrated notable anticancer effects in triple-negative breast cancer, inducing 32.6% apoptosis in MDA-MB-231 cells compared to just 10.6% in controls [235]. These formulations exhibited sustained drug release, enhanced cellular uptake, and favorable in vivo biodistribution in BALB/c mice.

For resveratrol, SLNs have similarly improved therapeutic efficacy. Resveratrol-loaded SLNs (200 nm) based on stearic acid increased apoptosis in MDA-MB-231 breast cancer cells through modulation of key apoptotic proteins—increasing Bax and decreasing Bcl-2, cyclin D1, and c-Myc expression [236]. More advanced versions of SLNs, such as nanostructured lipid carriers (NLCs), have further expanded delivery options for these natural compounds. NLCs contain a mixture of solid and liquid lipids, which creates imperfections in the crystal structure, allowing for higher drug loading and improved stability compared to traditional SLNs. Resveratrol-loaded NLCs have demonstrated particular promise for breast cancer therapy. In a study examining local delivery to mammary tissues, resveratrol-loaded NLCs delivered via microneedle arrays showed increased permeation of resveratrol into the skin, significantly improving resveratrol’s bioavailability compared to conventional oral administration [237]. The anticancer activity of resveratrol was enhanced in MDA-MB-231 breast cancer cell lines when delivered through these NLCs, resulting in improved cellular internalization compared to pure resveratrol. This approach represents an effective strategy for localized delivery of resveratrol in breast cancer therapy [237,238].

The improved efficacy of SLNs and NLCs for delivering natural compounds can be attributed to their ability to overcome key limitations of these phytochemicals. These lipid-based systems enhance the biocompatibility, biodegradability, and solubility of compounds like curcumin and resveratrol, while also improving their permeability and shelf life [238]. The selection of appropriate lipid components and preparation methods is crucial for optimizing the physicochemical properties and therapeutic efficiency of these nanocarriers. When properly formulated, SLNs and NLCs can deliver anticancer compounds to tumors with high precision and efficacy, enhancing cellular uptake by cancer cells and improving overall therapeutic outcomes [237].

#### 4.2.4. Polymeric Nanoparticles

Polymeric nanoparticles have emerged as promising drug delivery systems for enhancing the therapeutic potential of natural compounds with known anticancer activity, such as rhein, curcumin, and resveratrol. Their poor bioavailability, instability, and limited solubility have been major challenges, which nanotechnology-based approaches aim to overcome.

In the case of rhein, PLGA nanoparticles (~114 nm) co-loaded with rhein and hesperidin (RH-NP) were shown to significantly modulate the tumor immune microenvironment in triple-negative breast cancer. These nanoparticles suppressed cancer-associated fibroblasts (CAFs) and inhibited CCL2 secretion by cancer-associated adipocytes (CAAs), leading to enhanced cytotoxic T-cell infiltration and decreased levels of immunosuppressive cells, ultimately resulting in potent antitumor effects [239].

Curcumin, a polyphenolic compound with well-documented anticancer properties, was successfully encapsulated into mPEG-b-PMMA polymeric micelles, resulting in a more than 400-fold increase in solubility and improved stability, especially under alkaline conditions. These micelles exhibited dose-dependent cytotoxicity in several cancer cell lines, including SW-48 (colorectal), A2780 (ovarian), and HepG2 (hepatoma), highlighting their efficacy as curcumin nanocarriers [240].

Numerous nanoparticle-based systems have also been designed for resveratrol, addressing its low solubility and rapid metabolism. Gelatin nanoparticles (~294 nm) loaded with resveratrol enhanced cellular uptake, DNA damage, and apoptosis in NCI-H460 lung cancer cells via the modulation of key apoptotic markers (downregulation of Bcl-2; upregulation of p53, p21, and Bax) and induced G0/G1 cell cycle arrest [241]. Similarly, polymeric nanoparticles (~150 nm) achieved high encapsulation efficiency (74–98%) and exhibited enhanced cytotoxicity in both androgen-independent (DU-145, PC-3) and hormone-sensitive (LNCaP) prostate cancer cell lines compared to free resveratrol [242].

PEG-PLA nanoparticles loaded with resveratrol also demonstrated potent anticancer activity in CT26 colon cancer cells, inducing apoptosis, decreasing 18F-FDG uptake and oxidative stress in vitro, and reducing tumor growth while improving survival in tumor-bearing mice [243]. Transferrin-functionalized PEG-PLA nanoparticles (~150 nm) increased resveratrol uptake in glioma cells (C6 and U87), reduced tumor volume, accumulated in brain tumors, and extended survival in glioma-bearing rats, suggesting their potential as targeted glioblastoma therapy [244].

Resveratrol-loaded chitosan–TPP nanoparticles (~172–217 nm) offered pH-responsive release (increased at pH 6.5), retained antioxidant activity after UV exposure, and maintained antiproliferative activity in SMMC-7721 hepatocellular carcinoma cells while showing reduced toxicity in normal hepatocytes, supporting their applicability in targeted cancer therapy [245].

A hybrid system consisting of hyaluronic acid-conjugated mesoporous silica nanoparticles co-loaded with anti-miR21 and resveratrol showed a 3-fold higher tumor regression than free resveratrol and double the efficacy of non-targeted nanoparticles in CD44-overexpressing gastric cancer cells, through enhanced uptake and apoptosis induction [246].

In glioma models, mPEG-PCL nanoparticles (~135 nm) co-loaded with resveratrol and temozolomide acted synergistically by inhibiting the PI3K/Akt/mTOR signaling pathway, increasing pro-apoptotic protein expression, and delaying tumor progression in U87 xenograft mice [247].

Lastly, dual-targeted MDCA-based micelles physically encapsulating resveratrol enhanced internalization in HepG2 liver cancer cells and improved in vivo antitumor effects, including angiogenesis inhibition and apoptosis induction [248].

Collectively, these findings underline the versatility and efficacy of polymeric nanoparticles as delivery platforms for rhein, curcumin, and resveratrol in cancer treatment, offering improved pharmacokinetics, tumor targeting, and therapeutic outcomes.

#### 4.2.5. Metal-Based Nanoparticles

Metal-based nanoparticles have emerged as versatile delivery platforms for natural compounds with anticancer properties. These nanocarriers offer unique advantages, including surface functionalization capabilities, stimuli-responsive release, and in some cases, intrinsic therapeutic effects that complement the loaded compounds. For rhein delivery, magnetic nanoparticles have shown particular promise. Rhein-loaded heparin-coated magnetic nanoparticles significantly enhanced antitumor effects against HepG2 cells, reducing viability to approximately 10% compared to the free drug [249]. The magnetic properties of these carriers enable field-directed accumulation at tumor sites, enhancing therapeutic efficacy.

Gold nanoparticles represent another important class of metal-based carriers for these natural compounds. Studies have demonstrated that gold-conjugated resveratrol nanoparticles exhibit superior anti-invasive activity against MCF-7 breast cancer cells compared to free resveratrol by effectively inhibiting matrix metalloproteinase-9 (MMP-9), cyclooxygenase-2 (COX-2), and nuclear factor kappa B (NF-κB) expression [250]. The incorporation of targeting ligands further enhances the specificity of these nanocarriers. For instance, folate-targeted gold nanoparticles co-loaded with curcumin and docetaxel (FA-Cur + DTX-AuNPs) have shown enhanced inhibition of proliferation and the epithelial–mesenchymal transition (EMT) while inducing apoptosis in breast cancer cells [251]. Gold nanoparticles can also serve as active targeting drug delivery carriers when modified with hyaluronic acid (HA), which significantly increases the efficiency of compound utilization and prolongs retention time in vivo [252].

Zinc oxide (ZnO) nanoparticles have shown synergistic effects when combined with resveratrol. ZnO nanoparticles conjugated with trans-resveratrol enhance reactive oxygen species (ROS) activity and antioxidant properties through electron migration and mitochondrial membrane depolarization, resulting in significantly improved cancer cell death in PA1 ovarian cancer cells compared to free resveratrol [253]. This synergistic effect highlights how the intrinsic properties of metal nanocarriers can complement and enhance the therapeutic effects of the loaded compounds.

Iron oxide-based magnetic nanoparticles offer additional advantages for targeted delivery. Magnetic lignin/Fe_3_O_4_ nanoparticles loaded with resveratrol (AL/RSV/Fe_3_O_4_-NPs) demonstrated magnetic-field-enhanced accumulation and tumor inhibition in murine lung carcinoma, along with improved stability, sustained release, and reduced systemic toxicity [254]. The ability to externally direct these nanocarriers using magnetic fields enables precise targeting of tumor sites, potentially enhancing therapeutic efficacy while reducing off-target effects.

Surface modification of metal nanoparticles with hyaluronic acid has proven particularly effective for targeting CD44-overexpressing cancer cells. HA-modified curcumin nanocrystals (HA-Cur-NCs) have shown enhanced intracellular uptake in CD44-overexpressing MDA-MB-231 cells, superior anticancer effects in murine breast cancer models, and significantly extended half-life and mean residence time compared to free curcumin and unmodified curcumin nanocrystals [252,255]. This approach addresses the key limitations of natural compounds by improving their bioavailability and targeting capabilities.

Metal-based nanoparticles can be particularly effective for delivering natural compounds like curcumin, which has demonstrated anti-inflammatory and anticancer properties but suffers from poor bioavailability [256]. The use of nanocarriers enhances curcumin’s therapeutic properties by increasing its availability at tumor sites [256]. Similarly, resveratrol-loaded nanoparticles have shown the ability to inhibit breast cancer cell proliferation in vitro and target tumor-associated macrophages (TAMs) to suppress tumor growth in vivo [256].

The development of hybrid metal-based nanocarriers has further expanded delivery options. These systems combine the advantages of different materials to create multifunctional platforms with enhanced stability, targeting capabilities, and therapeutic efficacy. These advanced designs represent promising approaches for maximizing the therapeutic potential of natural compounds in cancer treatment.

Table 1 systematically presents these delivery systems based on nanoparticle type, targeting mechanisms, encapsulation efficiency, and therapeutic outcomes across various cancer models. By highlighting recent advances and persistent challenges, this overview offers valuable insights into the translational potential of these nanodelivery platforms for enhancing the anticancer efficacy of natural compounds while minimizing systemic toxicity.

Despite the promising therapeutic potential of rhein, resveratrol, and curcumin, their clinical application faces significant challenges related to poor bioavailability. Nanocarrier delivery systems offer solutions to these limitations through enhanced solubility, controlled release, and targeted delivery [165]. Different nanocarrier types exhibit varying efficacy profiles for delivering these natural compounds (Table 2).

### 4.3. Comparative Analysis of Nanocarrier Efficacy and Biocompatibility in Published Studies Involving Rhein, Curcumin, and Resveratrol

A comparative analysis of nanocarrier efficacy across published studies reveals that liposomal formulations generally offer superior protection against enzymatic degradation in biological fluids, while polymeric nanoparticles provide better controlled-release properties. Metal-based nanoparticles demonstrate advantages for targeted delivery using external stimuli but may present higher toxicity concerns compared to lipid-based systems. The future direction of nanocarrier development for these compounds focuses on several key areas. First, the development of multifunctional nanocarriers that combine the advantages of different materials shows promise for overcoming current limitations. Second, customization with targeting moieties such as antibodies or peptides can enhance specificity toward diseased tissues or cells, leading to higher concentrations at target sites and improved therapeutic outcomes [218]. Third, innovative approaches using nanoemulsions are being investigated to optimize the stability and biodistribution of compounds like resveratrol [224]. The choice of nanocarrier type significantly impacts encapsulation efficiency and therapeutic efficacy. Polymer nanoparticles are widely used due to their high encapsulation efficiency, which reduces the required carrier material and minimizes potential toxicity [217]. While synthetic nanoparticles like inorganic carriers have limitations including low encapsulation capacity and safety concerns, biologically derived carriers offer improved biological distribution, cellular uptake, and controlled drug release with higher biocompatibility [217]. However, obtaining bionic nanocarriers like protein-based systems and exosomes on a large scale remains challenging, limiting their commercial application.

When comparing the data reported in the literature from in vitro and/or in vivo studies investigating the antitumor efficacy of various compounds across different tumor cell lines, a clear difference can be observed between nanoformulated active substances and their free (non-formulated) counterparts.

Chang et al. demonstrated that treatment of MCF-7 tumor cells with rhein at a concentration of 1000 μg/mL (3.5 mM) resulted in cell survival rates of less than 10% [10]. The IC50 value of rhein was reported as 36.69 ± 9.77 μg/mL (129.1 ± 34.37 μM) for MCF-7/VEC cells and 30.66 ± 2.21 μg/mL (107.9 ± 7.7 μM) for MCF-7/HER2 cells. Lin and Zhen evaluated the antitumor activity of rhein lysinate (RHL), a salt formed with lysine to improve the aqueous solubility of rhein [264]. RHL was tested in vitro on MCF-7, SK-Br-3, and MDA-MB-231 tumor cell lines, both as a single agent and in combination with Taxol. When cells were treated with the RHL–Taxol combination, the IC50 values were 0.04 mM RHL and 0.16 μM Taxol—significantly lower than the IC50 values of 0.1 mM (RHL) and 1.2 μM (Taxol) when administered individually. In vivo studies were conducted using an MCF-7 breast tumor model in female Balb/c athymic mice, with treatment regimens of RHL (100 mg/kg, twice weekly for four weeks), Taxol (10 mg/kg, once weekly for two weeks), or the combination at the same doses. The observed tumor growth inhibition rates were 46.7%, 60%, and 85%, respectively. These findings highlight the important potentiating role of rhein in enhancing the antitumor effect of Taxol, while rhein alone showed only modest activity. Ping Shi et al. treated BEL-7402 hepatocellular carcinoma cells with various concentrations of rhein solubilized in water containing 0.1% DMSO. At a concentration of 200 μM, rhein inhibited cell proliferation by approximately 70% [265]. Changying Shi et al. developed telodendrimer-based nanocarriers encapsulating rhein, which were tested on Raji lymphoma cells over 72 h [266]. Cell viability ranged from 70 to 80% with Rhein-loaded nanocarriers. However, when the nanocarriers also contained doxorubicin (DOX), cell viability dropped to 20% at the highest administered concentration (1000 μg/mL). In vivo administration of a nanoformulated combination containing 10% DOX and rhein resulted in a tumor volume approximately 4.5 times smaller than that of the untreated control group and a survival time of 50 days, nearly twice that of the control group [266]. Haiyang Feng et al. developed solid lipid nanoparticles with 98.2 ± 6.7% rhein loading (Rh-SLNs); they showed a rapid burst release phase within 2 h, followed by sustained release [267]. After 48 h treatment of SW480 human colon carcinoma cells, free rhein resulted in approximately 80% cell viability, whereas Rh-SLNs showed enhanced cytotoxicity, with cell viability reduced to 50% at 35 μM and to 40% at twice that concentration [267]. Durdureanu-Angheluta et al. encapsulated rhein in the heparin shell of magnetic nanoparticles (MP-HP-Rh). The exposure of HepG2 hepatocellular carcinoma cells to a suspension containing 30 μM rhein for 24 h resulted in cell viability below 10%, compared to approximately 60% for free rhein at the same concentration. Since the other nanoparticle components exhibited proliferative effects, this nanoformulation was considered highly effective and potentially applicable to other cell lines and subsequent animal tumor models [249]. A recent study by Filipiuc et al. evaluated the effect of a liposomal rhein formulation (Lip-Rh) on MCF-7 cells following 72 h exposure. At 50 μM rhein, cell viability was 48% for the free compound and 20% for Lip-Rh [26]. Although stronger antitumor effects were observed at 75 and 100 μM, treatment with 50 μM caused only subtoxic effects on normal human gingival fibroblasts (HGFs), with cell viability around 80%. Higher concentrations of rhein showed increased cytotoxicity. Since unloaded liposomes have a proliferative effect due to their structural similarity to cell membranes, the Lip-Rh formulation shows promising potential for further preclinical evaluation in animal tumor models.

Studies have consistently shown that curcumin inhibits cancer cell proliferation in a dose- and time-dependent manner. Parte et al. showed that in CC531 colorectal cancer cells, curcumin reduced cell proliferation by more than 30% after 48 h of treatment, with higher doses (25–30 μM) reducing cell viability to less than 50% after 72 h [268]. The in vivo experiments of the same study proved that curcumin administration significantly reduced tumor volume of liver implants by 5.6-fold compared to controls. Similar antiproliferative effects have been observed by Bimonte et al. in MIA PaCa-2 pancreatic cancer cells and MDA-MB-231 breast cancer cells and by Termini et al. in prostate cancer cells [269,270,271].

To address curcumin’s poor bioavailability, researchers have developed various delivery systems with enhanced in vivo efficacy. Chen et al. studied the in vitro cytotoxic effects of free curcumin and curcumin-loaded pH-sensitive liposomes (LCCs) on HCT-116 cells and demonstrated a concentration-dependent inhibition of cell proliferation [223]. LCCs significantly enhanced the cytotoxicity of curcumin compared to the free compound. The IC50 values were 9.413 μM for free curcumin and 5.067 μM for LCCs, indicating that the liposomal carrier system improved the antiproliferative activity of curcumin and enhanced tumor cell growth inhibition. In vivo experiments on an orthotopic colon cancer model induced in C57BL/6 mice evaluated the antitumor efficacy of curcumin-loaded liposomes (LCCs) compared to free curcumin. LCCs intravenously injected via the tail vein at an equivalent dose of 2.5 mg/kg curcumin every 3 days for five times demonstrated a superior therapeutic effect, since the tumor volume in the group treated with free curcumin was approximately 1.6 times greater than that observed in the LCC-treated group. Additionally, LCC treatment completely prevented mortality during the 55-day study period, highlighting improved survival outcomes. The enhanced efficacy of LCCs was attributed to their improved stability, prolonged circulation time, and pH-sensitive CaCO_3_-based delivery, which facilitated more efficient drug release in the tumor microenvironment.

Bharmoria et al. investigated curcumin-loaded olive-oil-in-water nanoemulsions (Cur-NEs) for anticancer therapy [272]. Cur-NEs significantly reduced MDA-MB-231 breast cancer cell viability to below 40%, while maintaining ~80% viability in healthy L929 fibroblasts, indicating good selectivity. In contrast, gelatin-coated nanoemulsions (G-Cur-NEs) reduced viability in both cell types, suggesting higher overall cytotoxicity. These findings support Cur-NEs as a more selective formulation, while G-Cur-NEs may offer enhanced efficacy at the cost of increased toxicity to normal cells.

Sarkar et al. prepared curcumin-loaded solid lipid nanoparticles (CUR_SLNs), that showed potent antitumor activity against MDA-MB-231 breast cancer cells, with an IC50 of 0.85 mg/mL and 32.6% apoptosis induction [235]. In contrast, toxicity toward normal HEK293T cells was low: cell viability remained 97.6% at the IC50 dose, 81.0% at 8.5 mg/mL, and 69.7% even at the highest tested concentration (16.9 mg/mL). These results indicate strong selectivity for cancer cells and minimal toxicity to healthy cells. Biodistribution studies in mice confirmed accumulation in breast, brain, and spleen tissues, supporting CUR_SLNs as a promising, safe delivery system for targeted breast cancer therapy.

Sarkar et al. have developed curcumin-loaded polymeric micelles, which significantly reduced the viability and colony formation of A2780, HepG2, and SW-48 cancer cells in a dose-dependent manner [235]. The IC50 values for free CUR were 6.17 μg/ml (A2780), 4.82 μg/mL (HepG2), and 2.84 μg/mL (SW-48), while for CUR-loaded P2 micelles, the values were 6.27 μg/mL (A2780), 13.9 μg/mL (HepG2), and 5.76 μg/mL (SW-48). CUR-loaded micelles exhibited higher cytotoxicity than free CUR in A2780 cells at 20 μg/mL, likely due to the enhanced solubility and slow release of CUR. The lowest IC50 was found in SW-48 cells [235].

Kondath et al. have shown that curcumin-loaded gold nanoparticles (cAuNPs) exhibited potent, selective cytotoxicity against MCF-7 breast cancer cells, significantly reducing cell viability in a concentration, and time-dependent manner, down to 25% at 90 mg/mL after 72 h [273]. The cAuNPs contained curcumin in amounts equivalent to 1.75, 3.5, and 7 mg/mL. In contrast, treatment with free curcumin alone at the same concentrations resulted in only moderate cytotoxicity (viability remained 73% at 7 mg/mL), underscoring a synergistic effect of curcumin when conjugated to gold nanoparticles. Normal breast cells (HBL-100) maintained high viability (>90% at 48 h and 73% at 72 h with 90 mg/mL cAuNPs), confirming the biocompatibility of the formulation. These findings position cAuNPs as an effective and safe curcumin delivery system for targeted breast cancer therapy [273].

For resveratrol specifically, multiple delivery systems have been developed, including lipid nanocarriers, liposomes, emulsions, polymeric nanoparticles, and solid dispersions, each facilitating rapid absorption and increased plasma concentrations [173,212]. Resveratrol nanoformulations specifically exhibit advantages beyond enhanced bioavailability, including protection from environmental degradation, improved stability, controlled delivery, and selective cytotoxicity limited to cancer cells [214]. These formulations demonstrate slow release at injection sites, potentially reducing the risk of adverse effects while maintaining therapeutic efficacy. Similar benefits have been observed with curcumin and rhein nanoformulations, suggesting common advantages across different phytochemical compounds.

Wu et al. have demonstrated that resveratrol displayed significant antitumor effects [274]. In vitro tests on EJ human transitional cell carcinoma (TCC) cells treated with resveratrol at concentrations of 100, 150, and 200 μM for 1, 1.5, or 2 h daily over a 72 h period showed a clear dose- and time-dependent reduction in cell proliferation, with the highest inhibition observed at 200 μM . In vivo experiments on BALB/c-nude mice model with orthotopic bladder cancer showed significantly reduced bladder tumor weights in mice treated with 0.15 mg/kg resveratrol compared to controls. These findings confirm the antiproliferative and pro-apoptotic activity of resveratrol, supporting its therapeutic potential in bladder cancer [274].

A self-microemulsifying formulation developed by Jaisamut et al. combining curcumin and resveratrol (1:1 ratio) significantly enhanced their oral bioavailability and antitumor activity. Over 70% of curcumin and 80% of resveratrol were released within 20 min, forming oil-in-water droplets of 15–20 nm. The co-formulation showed stronger antioxidant effects and greater cytotoxicity against HT-29 colon cancer cells (IC_50_ = 18.25 µM) compared to curcumin (IC_50_ = 30.1 µM) or resveratrol alone (IC_50_ = 25.4 µM). In vivo, oral administration in rabbits increased plasma concentrations of curcumin and resveratrol by 10-fold and 6-fold, respectively, versus the unformulated combination [229].

Wang et al. have shown that resveratrol and its solid lipid nanoparticle formulation (Res-SLNs) demonstrated potent antitumor effects against MDA-MB-231 breast cancer cells [236]. Both free resveratrol and Res-SLNs reduced cell viability in a dose-dependent manner, with Res-SLNs showing significantly enhanced cytotoxicity (IC50 = 40.82 ± 3.92 µg/mL) compared to free resveratrol (IC_50_ = 72.06 ± 7.85 µg/mL). Apoptosis assays revealed a higher percentage of apoptotic cells following Res-SLN treatment (25.6%) than that following free resveratrol treatment (19.2%), indicating superior pro-apoptotic activity. Additionally, Res-SLNs induced more pronounced G0/G1 cell cycle arrest (25.5% vs. 19.2%) and more effectively inhibited cell migration and invasion than the free compound. These results highlight the improved antitumor efficacy of resveratrol when delivered via lipid-based nanoparticles [236].

Dana et al. tested liposomal resveratrol (L-RES), which proved enhanced antitumor activity in colorectal cancer (CRC) by targeting both tumor cells and cancer-associated fibroblasts (CAFs) [275]. In HT-29 cells (human colorectal adenocarcinoma), L-RES reduced cell viability to 94.7% at 62.5 μM, compared to 79.1% for free resveratrol. In MRC-5 fibroblasts (human lung fibroblasts), L-RES showed low cytotoxicity, maintaining 89.75% viability at the same concentration, indicating selective action toward the tumor microenvironment. In 3D co-culture tumor spheroids, L-RES significantly reduced spheroid growth and increased CRC sensitivity to 5-fluorouracil (5-FU, 5 μM) compared to controls. Additionally, L-RES downregulated CAF activation markers α-SMA and IL-6, inhibited CAF-induced cancer cell invasion (Boyden chamber and spheroid invasion assays), and was more effective than free resveratrol in suppressing tumor–stroma interactions. These findings support L-RES as a promising delivery system for CRC therapy through the dual targeting of cancer cells and tumor-supporting fibroblasts [275].

A recent study by the same authors emphasized the enhanced antitumor efficacy achieved by encapsulating resveratrol (RES) in poly (D,L-lactic-co-glycolic acid) nanoparticles (PLGA-RES, 178.4 ± 4.6 nm) and combining it with free sunitinib (SUNI), a tyrosine kinase inhibitor. In HT-29 colorectal adenocarcinoma cells, the combination at a fixed SUNI/RES ratio of 1:8, based on IC_50_ values of 14.4 µM and 110.9 µM, respectively, produced a synergistic reduction in cell viability, confirmed by combination index (CI) values < 1. In 2D cultures, PLGA-RES + SUNI reduced viability by ~25% and ~15% more than PLGA-RES or SUNI alone. In 3D tumor spheroids, the combination therapy significantly decreased spheroid volume and viability in a dose- and time-dependent manner (e.g., 16 + 128 µM SUNI + RES over 48 h), with pronounced disruption of spheroid structure. Compared to free drugs or unloaded nanoparticles, the nanoencapsulated RES formulation in combination with SUNI demonstrated the most potent antitumor effect, highlighting its promise as a co-delivery strategy for colorectal cancer therapy [276].

All nanoformulations discussed in this review, which exhibited superior antitumor efficacy compared to their corresponding free compounds, were primarily assessed in terms of cellular toxicity. These evaluations were conducted on both cancerous and normal cell lines, with parallel treatments using the free drug, the unloaded nanocarrier, and the nanoformulated agent. In most cases, unloaded carriers showed proliferative, nontoxic, or subtoxic effects on both cell types. Notably, carriers that closely mimic biological structures, such as liposomes, composed mainly of phospholipids and cholesterol, demonstrated superior cytocompatibility. Beyond in vitro safety, in vivo experiments further reinforced the therapeutic benefits of these nanoformulations, as most studies in tumor-bearing animal models reported increased survival rates following treatment. Moreover, nanoformulations with tumor-targeting capabilities significantly reduced off-target toxicity in healthy tissues and organs. Nevertheless, to advance these systems toward clinical application, comprehensive toxicological studies, encompassing acute, subchronic, and chronic toxicity, are essential to ensure their safety and regulatory compliance.

## 5. Conclusions and Future Directions

This review article offers a comprehensive and up-to-date overview of the antitumor potential of three well-studied natural compounds: rhein, curcumin, and resveratrol, focusing on their application in cancer therapy through advanced nanotechnology-based drug delivery systems. While previous reviews have often discussed these compounds individually or in broader phytochemical contexts, our work distinguishes itself by integrating detailed, comparative data from recent studies involving various nanocarriers, such as liposomes, polymeric nanoparticles, micelles, self-emulsifying systems, and others [8,37,277,278,279,280,281]. We highlight actual examples of improved pharmacokinetics, enhanced cellular uptake, synergistic effects in combination therapies, and superior tumor targeting achieved through nanoformulations, covering both in vitro and in vivo models across multiple cancer types. The rationale for selecting these three compounds lies in their demonstrated potency as natural anticancer agents and their strong potential for clinical translation if further investigated through well-designed preclinical and clinical studies. By critically analyzing these findings side by side, this review identifies formulation-specific advantages and limitations and proposes directions for translational research. Thus, it offers added value to the existing literature by consolidating dispersed data into a focused, evidence-based resource that supports the rational design of future nanoformulations for natural anticancer agents.

As demonstrated throughout this review, extensive research into the antitumor efficacy of the three natural compounds, rhein, curcumin, and resveratrol, has revealed a broad spectrum of biological activities mediated through multiple molecular pathways. These effects are highly context-dependent, varying according to factors such as cancer type and model, tumor stage, compound formulation, and route of administration. While numerous studies have reported promising outcomes, including marked antiproliferative and pro-apoptotic effects, translation into clinical practice requires adherence to established preclinical and clinical development frameworks as defined by regulatory authorities.

Importantly, these compounds have not been evaluated uniformly across all pharmacological levels. Rhein, in particular, remains less studied, likely due to its poor water solubility, which poses a significant barrier to parenteral formulation development. Among the current strategies utilized to improve the bioavailability and therapeutic index of these agents, nanotechnology-based drug delivery systems appear especially promising. These platforms allow for targeted delivery to tumor tissues and enhanced drug stability. However, challenges remain, including the need for high volumes of colloidal formulations to reach effective concentrations, as well as the requirement to independently assess the pharmacokinetic and pharmacodynamic profiles of both the active compound and the delivery vehicle. In conclusion, while curcumin, resveratrol, and rhein hold substantial potential as anticancer agents, further comprehensive studies are needed to optimize their formulation, evaluate their long-term safety and efficacy, and facilitate their integration into standard therapeutic regimens. Continued interdisciplinary efforts in medicinal chemistry, nanotechnology, and oncology will be essential to fully unlock their clinical value.

Studies on various nanocarriers loaded with rhein, curcumin, and resveratrol as natural-origin compounds have demonstrated significant effects in inhibiting cell proliferation and reducing tumor volume across different cancer types, including colorectal, pancreatic, breast, prostate, and many other cancers. However, to draw clear conclusions about the safety and efficacy of these formulations, systematic studies should be conducted, focusing on specific cancer types and underlying mechanisms. Future research should include clinical trials to determine whether these molecules can be safely administered as a monotherapy, in combination therapies, or as an adjuvant compound. Furthermore, optimizing delivery systems to maximize efficiency while minimizing toxicity will be crucial in providing safer and more effective therapeutic solutions.

## Figures and Tables

**Table 1:** Nanotechnology-enabled drug delivery systems of rhein, curcumin, and resveratrol in cancer therapy.

Nanocarrier Type	Compound	Results	Reference
**Liposomes**	**Rhein**	**Liposomal rhein:** - Reduces the required dose by 2.5× compared to free rhein in MCF-7 cells. - Exhibits low cytotoxicity in normal cells (enhanced tumor selectivity).	[26]
		**HA-modified liposomes (~190 nm):** - Improve cellular uptake, cytotoxicity, and anti-metastatic activity in 4T1 cells. - In vivo: reduce lung metastases, improve tumor targeting, and antitumor efficacy compared to free rhein and unmodified liposomes.	[11]
	**Curcumin**	**Liposomal curcumin (~100 nm):** - Strong antitumor activity in colorectal cancer (LoVo, Colo205) via apoptosis induction. - Shows synergism with oxaliplatin (4:1 ratio); outperforms oxaliplatin in vivo by reducing VEGF, IL-8, and CD31 expression.	[222]
		**Curcumin-loaded CaCO_3_-encapsulated liposomes (LCCs), pH-sensitive formulation:** - Enhances cytosolic delivery and lysosomal escape. - Superior antitumor efficacy in AOM/DSS-induced murine colorectal cancer model compared to standard liposomes.	[223]
	**Resveratrol/** **Paclitaxel**	**PEGylated liposomes co-encapsulating paclitaxel and resveratrol (~50 nm):** - Had improved bioavailability, tumor accumulation, and cytotoxicity in drug-resistant MCF-7/Adr cells. - Induced synergistic apoptosis and inhibit tumor growth in vivo without added systemic toxicity.	[158]
**Nanoemulsions**	**Curcumin**	**Curcumin nanoemulsion (44 nm):** - Reduced tumor index by 91.81% and tumor area by 89.95% in K14E6 transgenic mice. - Prevented microinvasive squamous cell carcinoma; downregulated Cdk4 gene expression.	[257]
	**Curcumin +** **Resveratrol**	**Self-microemulsifying formulation containing curcumin and resveratrol (15–20 nm droplets):** - Enhanced antioxidant activity and synergistic cytotoxicity against HT-29 cells (IC50 = 18.25 μM). - Increased oral bioavailability: 10-fold for curcumin and 6-fold for resveratrol.	[229]
	**Curcumin/5-fluorouracil**	**Curcumin/5-fluorouracil co-loaded agarose–chitosan nanoemulsion:** - Showed high drug loading (85.6%) and pH-sensitive release (up to 44% difference). - Enhanced cytotoxicity against MCF-7 cells; maintains carrier biocompatibility.	[258]
**Solid Lipid** **Nanoparticles (SLNs)**	**Curcumin**	**Curcumin-loaded solid lipid nanoparticles (311 nm):** - Induced 32.6% apoptosis in MDA-MB-231 triple-negative breast cancer cells (vs. 10.6% in controls). - Showed sustained release, improved cellular uptake, and favorable biodistribution in mice.	[235]
	**Resveratrol**	**Resveratrol-loaded SLNs (200 nm):** - Promote apoptosis in MDA-MB-231 cells by increasing Bax and reducing Bcl-2, cyclin D1, and c-Myc expression.	[236]
**Polymeric** **Nanoparticles**	**Rhein**	**PLGA nanoparticles (~114 nm) co-loaded with rhein and hesperidin (RH-NP):** - Suppress CAFs and CCL2 secretion from CAAs in TNBC. - Enhance cytotoxic T-cell infiltration, reduce immunosuppressive cells. - Exhibit promising antitumor activity via immune modulation.	[239]
	**Curcumin**	**Curcumin-loaded mPEG-b-PMMA polymeric micelles:** - Improve curcumin solubility 400× and stability (especially in alkaline pH). - Show dose-dependent cytotoxicity in SW-48, A2780, and HepG2 cancer cells. - Effective nanocarrier candidate for curcumin.	[240]
	**Resveratrol**	**Resveratrol-loaded gelatin nanoparticles (294 nm):** - Enhance uptake, DNA damage, and apoptosis in NCI-H460 lung cancer cells. - Downregulate Bcl-2; upregulate p53, p21, Bax; arrest cell cycle in G0/G1 phase.	[241].
		**Polymeric nanoparticles (150 nm) loaded with trans-resveratrol achieved high encapsulation efficiency (74–98%):** - Enhanced cytotoxicity (10–40 µM) vs. androgen-independent and hormone-sensitive prostate cancer lines (DU-145, PC-3, LNCaP).	[242]
		**Resveratrol-loaded PEG-PLA polymer nanoparticles:** - Reduce CT26 colon cancer cell number to 5.6% and colony formation to 6.3%. - Induce apoptosis, reduce 18F-FDG uptake and ROS. - Improve survival in tumor-bearing mice.	[243]
		**Transferrin-modified PEG-PLA nanoparticles loaded with resveratrol (150 nm diameter):** - Increased uptake in glioma cells (C6, U87), enhanced cytotoxicity. - Accumulate in brain tumors, reduce tumor volume, prolong survival. - Potential for glioblastoma targeting.	[244]
		**Resveratrol-loaded chitosan–TPP nanoparticles (~172–217 nm):** - Improve solubility, pH-responsive release (higher at pH 6.5). - Maintain antiproliferative effect in SMMC-7721 cells with low toxicity to normal hepatocytes. - Preserve antioxidant activity post-UV.	[245]
		**Hyaluronic acid-conjugated mesoporous silica nanoparticles co-delivering anti-miR21 and resveratrol:** - Show 3× higher tumor regression vs. free resveratrol. - 2× better efficacy than non-targeted NPs in CD44+ gastric cancer cells. - Enhance uptake and apoptosis.	[246]
		**mPEG-PCL nanoparticles co-loaded with resveratrol and temozolomide (135 nm):** - Synergistically inhibit PI3K/Akt/mTOR signaling in U87 glioma model. - Increase pro-apoptotic protein expression, delay tumor growth in xenografts.	[247]
		**Resveratrol (RSV) encapsulated in MDCA-based micelles:** - Improves HepG2 cell internalization and in vivo antitumor effects. - Induces apoptosis and angiogenesis inhibition.	[248]
**Metal-based** **Nanoparticles**	**Rhein**	**Rhein-loaded heparin-coated magnetic nanoparticles:** - Reduced HepG2 tumor cell viability to ~10%. - Stronger antitumor effect than free rhein.	[249,259]
	**Curcumin**	**Folate-targeted gold nanoparticles co-loaded with curcumin and docetaxel (FA-Cur + DTX-AuNPs):** - Inhibited proliferation and EMT in MCF-7 and MDA-MB-231 cells. - Induced apoptosis, upregulated Bak, Bid, E-cadherin, p27KIP1. - Downregulated Bcl-xL, Cyclin D1, N-cadherin, Snail. - Synergistic effects, enhanced uptake, improved docetaxel efficacy.	[251]
	**Resveratrol**	**Gold-conjugated resveratrol nanoparticles:** - Superior anti-invasive activity in MCF-7 breast cancer cells. - Inhibited MMP-9, COX-2, NF-κB expression.	[250]
		**ZnO nanoparticles conjugated with trans-resveratrol:** - Enhanced ROS production and mitochondrial depolarization. - Increased expression of pro-apoptotic proteins (Bax, caspase-9) and decreased Bcl-2 expression. - Greater cancer cell death in PA1 ovarian cancer vs. free resveratrol.	[253]
		**Magnetic lignin/Fe_3_O_4_ nanoparticles with resveratrol (AL/RSV/Fe_3_O_4_-NPs, ~160 nm):** - Magnetic field-enhanced tumor targeting and accumulation. - Inhibited murine lung carcinoma growth. - Improved stability, sustained release, and reduced systemic toxicity.	[254]

**Table 2:** Bioavailability and delivery improvements.

Cancer Type Targeted	Natural Compound Used and Nanocarrier Type	Combination Therapy Approach	Mechanism of Action	Drug Delivery Challenges Addressed	Reference
Breast, colorectal, pancreatic, ovarian, prostate, lung, cervical, liver cancers and chronic myeloid leukemia	Curcumin Polymer-based nanoparticles	Combinations of curcumin with antitumor therapy for synergistic effects and overcoming drug resistance	Curcumin-loaded nanoparticles induce apoptosis, inhibit cell proliferation, prevent angiogenesis, disrupt signaling pathways (NF-κB/STAT3), and overcome multidrug resistance in tumor cells	Curcumin has poor bioavailability, low water solubility, and low stability. Polymer-based nanoparticles effectively overcome these drawbacks while enabling combination therapy with classic antitumor drugs	[260]
Drug-resistant breast cancer (MCF-7/Adr cells)	Resveratrol co-encapsulated with paclitaxel—PEGylated liposomes	Combining resveratrol with paclitaxel in PEGylated liposomes to achieve a synergistic effect	Overcoming drug resistance through co-therapy with resveratrol and paclitaxel	Improves bioavailability and tumor retention of resveratrol and paclitaxel in vivo	[158]
Primary breast cancer	Polyphenols, including from pomegranate extract, curcumin, green tea catechins (especially EGCG), resveratrol, quercetin, and tannic acid Nanoparticles for polyphenol delivery	Combination of polyphenols with chemotherapeutic drugs in nanoparticles for synergistic effects against breast cancer cells	Polyphenol-loaded nanoparticles inhibit tumor cell growth by inducing apoptosis, inhibiting metalloproteinases, suppressing the NF-κB pathway, and inducing cell cycle arrest	The low bioavailability of polyphenols limits their pharmacological potential	[261]
Breast cancer (MDA-MB-231 cells)	Resveratrol Solid lipid nanoparticles	Resveratrol loaded into solid lipid nanoparticles	The obtained nanoparticles induce apoptosis by increasing the Bax/Bcl-2 ratio and inhibit proliferation by downregulating cyclin D1 and c-Myc expression	The poor water solubility, rapid metabolism, and short plasma half-life of resveratrol were overcome using solid lipid nanoparticles	[236]
Breast, pancreatic, and prostate cancer	Resveratrol Gold nanoparticles conjugated with resveratrol, encapsulated with gum arabic	Resveratrol combined with gold nanoparticles for synergistic antitumor effects	Gold nanoparticles conjugated with resveratrol induce apoptosis via nuclear condensation, cell shrinkage, fragmentation, and cellular dysfunction in tumor cells	The nanocarrier system improves the bioavailability and stability of resveratrol for optimal delivery to tumor cells	[262]
Colorectal cancer	Polyphenolic compounds including resveratrol and curcumin Microsponges, nanoparticles, nanocapsules, nanovesicles, and lipid nanocarriers	Combinations of polyphenols with hemicellulose-based carriers to enhance therapeutic effects	Nanoparticles improve the bioavailability of polyphenols, enhancing their anti-inflammatory and anticancer effects by inducing apoptosis, inhibiting NF-κB, and reducing oxidative stress	Low bioavailability, limited bioaccessibility, instability during digestion, low solubility, and rapid degradation of polyphenolic compounds	[263]
